# Arctic pathways of Pacific Water: Arctic Ocean Model Intercomparison experiments

**DOI:** 10.1002/2015JC011299

**Published:** 2016-01-08

**Authors:** Yevgeny Aksenov, Michael Karcher, Andrey Proshutinsky, Rüdiger Gerdes, Beverly de Cuevas, Elena Golubeva, Frank Kauker, An T. Nguyen, Gennady A. Platov, Martin Wadley, Eiji Watanabe, Andrew C. Coward, A. J. George Nurser

**Affiliations:** ^1^ National Oceanography CentreSouthamptonUK; ^2^ Alfred Wegener InstituteBremerhavenGermany; ^3^ Woods Hole Oceanographic InstitutionFalmouthMassachusettsUSA; ^4^ Institute of Computational Mathematics and Mathematical Geophysics, Siberian Branch of Russian Academy of SciencesNovosibirskRussia; ^5^ Department of Mathematics and MechanicsNovosibirsk State UniversityNovosibirskRussia; ^6^ Massachusetts Institute of TechnologyCambridgeMassachusettsUSA; ^7^ School of MathematicsUniversity of East AngliaNorwichUK; ^8^ Japan Agency for Marine‐Earth Science and TechnologyKanagawaJapan

**Keywords:** Arctic Ocean, Beaufort Gyre, Pacific Water, ocean dynamics, wind forcing

## Abstract

Pacific Water (PW) enters the Arctic Ocean through Bering Strait and brings in heat, fresh water, and nutrients from the northern Bering Sea. The circulation of PW in the central Arctic Ocean is only partially understood due to the lack of observations. In this paper, pathways of PW are investigated using simulations with six state‐of‐the art regional and global Ocean General Circulation Models (OGCMs). In the simulations, PW is tracked by a passive tracer, released in Bering Strait. Simulated PW spreads from the Bering Strait region in three major branches. One of them starts in the Barrow Canyon, bringing PW along the continental slope of Alaska into the Canadian Straits and then into Baffin Bay. The second begins in the vicinity of the Herald Canyon and transports PW along the continental slope of the East Siberian Sea into the Transpolar Drift, and then through Fram Strait and the Greenland Sea. The third branch begins near the Herald Shoal and the central Chukchi shelf and brings PW into the Beaufort Gyre. In the models, the wind, acting via Ekman pumping, drives the seasonal and interannual variability of PW in the Canadian Basin of the Arctic Ocean. The wind affects the simulated PW pathways by changing the vertical shear of the relative vorticity of the ocean flow in the Canada Basin.

## Key Points

- Pathways of the Arctic Pacific Water are investigated in ocean models
- Variability of the Pacific Water due to wind is examined
- Mechanisms of the Pacific Water variability are suggested

## 1 Introduction

According to long‐term moorings deployed in Bering Strait in 1991, 1998–2011, the annual mean water transport from the northern Bering Sea into the Arctic Ocean through the strait varies from year to year and is ∼0.7–1.1 Sv or ∼22–35 × 10^3^ km^3^ a^−1^ (1 Sv = 10^6^ m^3^ s^−1^ = 31,536 km^3^ a^−1^) [*Woodgate et al*., 2012]. This inflow brings Pacific Water (PW) to the Arctic Ocean, supplying the ocean with ∼2030–3500 km^3^ a^−1^ of fresh water, referenced to a salinity of 34.8 [*Woodgate et al*., 2006, 2012]. The corresponding heat inflow from the Pacific Ocean to the Arctic is ∼3–6 × 10^20^ J a^−1^ of heat (calculated relative to −1.9°C water temperature) [e.g., *Woodgate et al*., 2010]. The *UNESCO* [1986] practical salinity unit scale (PSU) convention is used through the text (units are omitted for simplicity) to be consistent with the historical observational freshwater fluxes in Bering Strait and the observed Arctic liquid freshwater content [e.g., *Woodgate et al*., 2006; *Rabe et al*., 2011, 2014] and also with the UNESCO equation of state featured in the models participating in this study.

**Figure 1:**
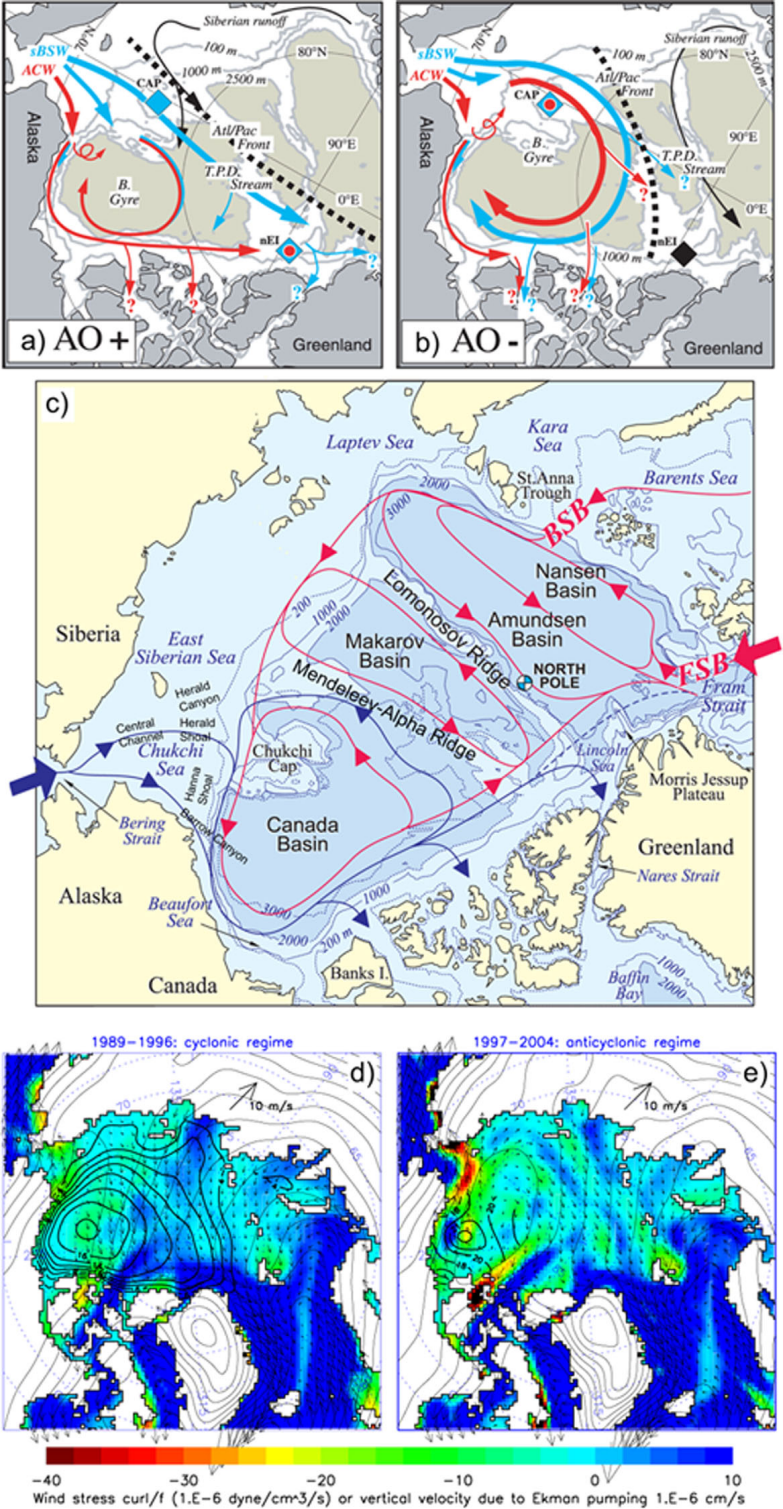
Schematic of Pacific Water (PW) circulation at 50–150 m depth adapted from (a,b) *Steele et al*. [2004] and (c) *McLaughlin et al*. [2002], respectively. Panels (a) and (b) show upper ocean circulation for high and low AO indices respectively (after *Steele et al*. [2004]). Blue and red arrows depict PW of different origin: Alaskan Coastal Water (ACW) and summer Bering Sea Water (sBSW). Thick broken line shows position of the Atlantic‐Pacific Front. The difference between PW circulations for different regimes is the amount of water transported to the North Atlantic via Canadian and Fram Straits: PW exits the Arctic Ocean through Fram Strait and the straits of Canadian Arctic Archipelago during high AO index, and it mainly flows out through the straits of the Canadian Arctic Archipelago during low AO indices. In (c) blue arrows show PW circulation after *McLaughlin et al*. [2002]. Red arrows mark Atlantic Water pathways with the Fram Strait Branch (FSB) and Barents Sea Branches (BSB) of the Atlantic flow. For the rest of abbreviations, see the citations above. Panels (d) and (e) show changes in the wind (arrows), wind stress curl (color), and freshwater content (thick solid lines) between 1989–1996 and 1997–2004. The thin solid lines are the sea level pressure contours.

Oceanic exchange through Bering Strait was a subject of several observational programs [*Coachman et al*., 1975; *Woodgate et al*., 2005b, 2010] and also has been examined in the Arctic Ocean Model Intercomparison Project (AOMIP) [*Clement Kinney et al*., 2014]. Circulation of PW on the shelf of the Chukchi Sea has been investigated in detail both by hydrographic observations [*Aagaard et al*., 1985; *Weingartner et al*., 2005; Woodgate et al., 2005a] and modeling studies [*Proshutinsky*, 1986; *Overland and Roach*, 1987; *Winsor and Chapman*, 2004; *Spall*, 2007; *Panteleev et al*., 2010; *Hu and Myers*, 2013; *Timmermans et al*., 2014]. A recent comprehensive overview of PW pathways is given by *Timmermans et al*. [2014], with the focus on the summer mode waters of the Pacific inflow, the lighter Alaskan Coastal Water (ACW) and denser summer Bering Sea Water (sBSW). They described the following three available routes for the summer PW across the Chukchi Sea (Figure 1): (1) in the western Chukchi Sea sBSW flows northward into the northern Canada Basin through the Herald Canyon and (2) also transverses the central Chukchi shelf, crossing the Herald Shoal and the Hanna Shoal via the Central Channel; (3) in the eastern Chukchi Sea, both ACW and sBSW mode waters enter the Beaufort Sea through the Barrow Canyon as the Beaufort shelf‐break jet—an eastward flow along the edge of the Beaufort Sea shelf [*Mountain et al*., 1976; *Nikolopoulos et al*., 2009; *von Appen and Pickart*, 2012].

Observational and modeling studies [e.g., *von Appen and Pickart*, 2012; *Hu and Myers*, 2013; *Timmermans et al*., 2014] have suggested geostrophic balance as the main dynamical control of the PW flow through the Chukchi Sea, while emphasizing the role of eddies in the offshore transport of PW in the Beaufort Sea and the effect of the baroclinic instabilities on the eastward extent of the Beaufort shelf‐break jet.

As it crosses the shallow Chukchi Sea, PW cools by ∼0.7°C and loses ∼0.6 × 10^20^ J a^−1^ of heat to the atmosphere [e.g., *Woodgate et al*., 2005b]. It then enters the Canada Basin of the Arctic Ocean (Figure 1). Although detailed trajectories of the PW modes are not known, observations show that in the Canada Basin, a large fraction of PW (50–100% of the water column) occupies the depth range between approximately the surface and 250 m; below this depth the water mass is heavily diluted [*McLaughlin et al*., 2004; *Carmack et al*., 2008; *Yamamoto‐Kawai et al*., 2008] due to mixing with the Atlantic Waters occupying water column depths between 200 and 800 m [e.g., *McLaughlin et al*., 2009]. The Beaufort Gyre may extend down into the Atlantic Water layer, as suggested by dynamic height analysis that shows a weak anticyclonic flow in the Canada Basin at ca. 400 m depth [e.g., *McLaughlin*, *et al.*, 2009]. In the Makarov Basin and above the Lomonosov Ridge, the thickness of the PW layer decreases to less than 150 m with the PW fraction decreasing to less than 60% [*Ekwurzel et al*., 2001; *Jones and Anderson*, 2008; *Alkire et al*., 2007].

The freshwater fraction of PW contributes significantly to the formation of a strongly stratified Arctic halocline [*Aagaard and Carmack*, 1989; *Steele et al*., 2004; *Anderson et al*., 2013]. The Arctic halocline constitutes the Arctic subsurface waters and resides beneath the Arctic fresh surface mixed layer, separating the surface waters from the warm and salty Arctic upper intermediate waters of Atlantic origin, hereafter the Atlantic Water. The Atlantic Water enters the Arctic Ocean from the North Atlantic and Nordic Seas through Fram Strait and the western Barents Sea (Figure 1) [e.g., *Carmack et al*., 2015]. The Arctic halocline hinders the transfer of the Atlantic Water heat upward to the upper layers and to the base of sea ice [e.g., *Polyakov et al*., 2010]. The heat carried by the summer PW [*Shimada et al*., 2001, 2006; *Steele et al*., 2004] can also melt a significant portion of Arctic Sea ice if rapidly released to the surface [*Woodgate et al*., 2010, 2012; *Rainville and Woodgate*, 2009].

One critical question is whether wind‐driven spin‐up of the Beaufort Gyre can increase the vertical shear in the upper ocean velocity, thin the halocline and allow vertical heat transfer from the Atlantic Water to the base of the sea ice and so substantially reduce sea ice cover [e.g., *Giles et al.*, 2012]. Another question is whether the recently observed warmer, stronger Bering Strait inflow can increase oceanic heat flux into the central Arctic Ocean and accelerate sea ice retreat [*Woodgate*, *et al.* 2010, 2012]. The answers to these questions require knowledge of the PW pathways and the impact of PW on the stratification in the Arctic Ocean.

Different circulation patterns of PW in the Arctic Ocean have been inferred from water properties by *Jones* [2001], *McLaughlin et al*. [2002], and *Steele et al*. [2004]. The latter two publications suggested different circulation pathways for the winter and summer PW. *McLaughlin et al.* [2002] proposed a cyclonic (counter‐clockwise) schematic for the flow of the winter PW in the Canada Basin with the watermass exiting the Canada Basin along the northern shelf of the Canadian Arctic Archipelago. On the other hand, *Steele et al.* [2004] analyzed hydrographic observations in the Arctic Ocean and suggested an anticyclonic (clockwise) circulation of the summer PW in the basin and a weak cyclonic PW flow along the Alaskan shelf. They also commented on the widespread presence of the winter mode of PW (the winter Bering Sea Water) in the Canada and Makarov basins, but did not discuss pathways of this watermass (Figure 1). The PW circulation and thickness of the PW layer are subject to temporal variability [*McLaughlin et al*., 2002; *Morison et al*., 2012] depending on atmospheric circulation [*Proshutinsky et al*., 2002; *Steele et al*., 2004; *Timmermans et al*., 2014]. The fact that the inferred circulation schemes differ significantly from study to study demonstrates very well the level of uncertainty in our understanding of the PW circulation in the Arctic Ocean and motivates our analysis using results from numerical models.

The lateral extent of PW across the Canada Basin and the Makarov Basin (Figure 1) is thought to be colocated with the Transpolar Drift, and in the late 1980s and early 1990s retreated from being aligned with the Lomonosov Ridge to being above the Mendeleev‐Alpha Ridge [e.g., *McLaughlin et al*., 1996; *Steele and Boyd*, 1998]. The displacement was linked with a change of atmospheric circulation over the Arctic Ocean [e.g., *McLaughlin et al*., 2002; *Steele et al*., 2004] from anticyclonic in the 1980s to cyclonic in the 1990s [e.g., *McLaughlin et al*., 2002]. It was also linked with the changes in the Arctic Oscillation (AO) index from being positive in the late 1980s to earlier 1990s to becoming neutral in the late 1990s and beginning of 2000s [e.g., *Steele et al*., 2004], although some later studies questioned the robustness of this hypothesis [e.g., *Swift et al*., 2005].

The 2003–2005 return of the frontal zone between Canadian Basin and Eurasian Basin polar waters to the pre‐1990s near‐climatological conditions and a partial return of PW into the Makarov Basin observed in 2003–2006 were accompanied by the shift of the Transpolar Drift from the Mendeleev‐Alpha Ridge toward the Lomonosov Ridge [*Alkire et al*., 2007]. The changes were attributed to changes in the atmospheric circulation [*Morison et al*., 2006] and resulted in a greater spread of PW through the Canada and Makarov Basins. Based on the data from the Environmental Working Group (EWG) Atlas [*Arctic Climatology Project*, 1997, 1998], the North Pole Environmental Observatory (NPEO) hydrographic surveys, and the SCICEX cruises, *Morison et al*. [2006] argued that baroclinic adjustment in the upper 500 m of the ocean could modify the effect of the wind and delay the oceanic response by 3–7 years. Using long‐term moorings, *Woodgate et al*., [2012] found a 50% increase in the PW inflow (from ∼0.7 Sv in 2001 to 1.1 Sv in 2011) through Bering Strait between 2001 and 2011; this increased inflow, if sustained, is able to make a significant impact on the Arctic subsurface waters. These and other studies indicate that the variability of PW in the Arctic Ocean is more complex than previously assumed and that the important question “What drives variations of the PW extent on intra‐annual to decadal scales?” has not been fully answered.

The present study is motivated by the questions formulated above and is driven by several hypotheses. We expect that the strength and patterns of PW circulation in the Arctic Ocean are highly variable and depend on at least three major factors, namely:

1. The sea level gradient between the Pacific and Atlantic Oceans as a mechanism for driving the inflow of PW through Bering Strait—a hypothesis first proposed by *Shtokman* [1957] and then by *Coachman et al*. [1975], *Stigebrandt* [1984], *Proshutinsky* [1986], and *Woodgate et al*. [2005b, 2010]. Changes of this gradient regulate the intensity of the inflow and volume of PW waters entering the Arctic Ocean via Bering Strait, but local winds are also important.
2. Basin‐scale atmospheric circulation [e.g., *Proshutinsky and Johnson*, 1997; *Steele et al*., 2004] with significant influence of local winds on synoptic time scales [e.g., *Coachman et al*., 1975; *Aagaard et al*., 1985; *Roach et al*., 1995; *Woodgate et al*., 2005b].
3. Sea ice conditions which influence both rates of air‐ocean momentum transfer and ocean stratification on seasonal and interannual scales [e.g., *Gudkovich*, 1961; *Aagaard and Carmack*, 1989; *Rampal et al*., 2009].

Investigation of how these multiple factors influence the PW dynamics based on observational data alone is difficult because extensive long‐term measurements with high spatial resolution are required. Besides, because of the complexity of the PW dynamics due to the coinfluence of the factors mentioned above, it is difficult to clearly separate underlying physical processes using currently available observational data.

To investigate PW dynamics in this paper, the results from regional and global models obtained in the coordinated AOMIP experiments, and in the follow‐up research under the umbrella of the Forum for Arctic Modelling and Observational Synthesis (FAMOS), were analyzed with the aim of answering the above questions. Six regional and global Ocean General Circulation Models (OGCM, Table 1) were employed in this study to examine PW pathways in the Arctic and the mechanisms of PW variability.

**Table 1:** Models Used in the Study

Model	ORCA1	ICMMG	NAOSIM	COCO	ECCO2	OCCAM
Domain	Global	Regional (Arctic and North Atlantic from 20°S)	Regional	Regional (Arctic and North Atlantic)	Regional nested from ∼55°N	Global
Resolution in the Arctic^a^ (km)	37	35	28	25	23	8
Vertical coordinate	z	z	z	σ‐z	σ‐z	z
Model levels	75	33	30	28	50	66
Bering Strait model representation	Fully represented three model cells across	Open boundary three model cells across	Fully represented four model cells across	Open boundary three model cells across	Fully represented five model cells across	Channel model eight model cells across
Bering Strait exchange	Unconstrained	Prescribed Barotropic inflow of 0.8 Sv	Prescribed Barotropic inflow of 0.8 Sv	Prescribed seasonally varying inflow, mean 0.8 Sv	Unconstrained	Unconstrained
Sea ice dynamics	EVP	EVP	VP	EVP	VP	EVP
Sea ice thermodynamics	2 layer Semtner	CICE 3.14	2 layer Semtner	0 layer Semtner	7 thickness categories	2 layer Semtner
Vertical mixing	TKE	Richardson number	None	NK	KPP	KPP
Advection	TVD	Linear forward	FCT	UTOPIA	Daru and Tenod	Split‐QUICK
Atmospheric forcing	NCEP/NCAR	NCEP/NCAR	NCEP/NCAR	NCEP/NCAR	JRA25	NCEP/NCAR
Wind	6‐Hourly	6‐Hourly	Daily	Daily	6‐Hourly	6‐Hourly
Turbulent fluxes	6‐Hourly	6‐Hourly	Daily	Daily	6‐Hourly	6‐Hourly
Solar radiation	6‐Hourly	6‐Hourly	Daily	Daily	6‐Hourly	6‐Hourly
Runoff	Monthly (R‐AN)	Monthly (R‐AN)	Monthly (PL03)	Monthly (PL03)	Monthly (R‐AN)	Part of restoring
Analyzed period	1989–2007	1989–2007	1989–2007	1989–2007	1992–2007	1989–2007
Spin‐up	1948–1988 (41 years)	1948–1988 (41 years)	1948–1988 (41 years)	1979–1988 (20 years)^b^	None	1985–1988 (4 years)
Initial ocean T&S fields	PHC2.1^c^	PHC3.0	PHC2.1	PHC3.0	PHC3.0	PHC2.1^c^
Initial sea ice	DRAKKAR^d^	Sea ice free	End of 50 year spin‐up	Sea ice free	PIOMAS^e^	NSIDC/*Romanov* f
Surface salinity restoring time scale	PHC2.1^c^ 180 days	None	None	None	None	PHC2.1^c^ 40 days

a The lowest model resolution in the Arctic Ocean is given because of nonuniform model grids.

b Prior to the 1979–1988 integration, COCO was spun‐up for 10 years with repeated 1979 forcing.

c Uses PHC 2.1 in the Arctic, merged with the World Ocean Atlas Climatology globally.

d Initial sea ice fraction and thickness adapted from the DRAKKAR ORCA025 simulations [*Barnier et al*., 2006].

e Initial sea ice fraction and thickness were from the from the Pan‐Arctic Ice Ocean Modeling and Assimilation System (PIOMAS) [*Schweiger et al*., 2011].

f Initial sea ice fraction was from National Snow and Data Center (NSIDC) data set [*Comiso and Nishio*, 2008] and sea ice thickness was from *Romanov* [1995] data set.

TKE, turbulent kinetic energy mixing scheme [*Blanke and Delecluse*, 1993]; KPP profile as in *Large et al*. [1994]; Richardson number scheme follows [*Pacanowski and Philander*, 1981]; FCT, flux corrected transport [*Gerdes et al*., 2008]; JRA, Japanese Atmospheric Re‐Analysis (wind from European Centre for Medium Range Weather Forecasting ECMWF ERA Reanalysis); NCEP, National Centers for Environmental Prediction/National Center for Atmospheric Research Re‐Analysis; NCEP/NCAR, National Centers for Environmental/National Center for Atmospheric Research Re‐Analysis; PHC2.1/3.0, Polar Hydrography Centre Climatology, version 2.1/3.0; PL03, Climatological runoff [*Prange and Lohmann*, 2003]; R‐AN, R‐ArcticNET climatological runoff data set [*Lammers et al*., 2001]; CICE, Los Alamos Sea ice model; EVP, sea ice dynamics based on the elastic‐viscous‐plastic rheology; VP, sea ice dynamics based on the viscous‐plastic rheology; NK, the turbulence closure mixed layer scheme of Noh and Kim [1999].

This paper is structured as follows: section 2 introduces the key features of the models setup, formulates hypotheses and conditions for the numerical experiments, and identifies parameters to be analyzed and compared with available observations. This section also describes diagnostics used for the analysis. Results from the model experiments and comparisons between models and observations are presented in section 3. Sections 4 and 5 discuss PW pathways, and their spatial and temporal variability and mechanisms. A summary of the work and conclusions from the study are given in section 6. Appendix A gives details on model configurations, including forcing, initial, and boundary conditions. Appendix B lists calculations used in diagnosing model output.

## 2 Experimental Setup

### 2.1 Models

The key components of six coupled sea ice‐ocean models employed in the Pacific Water model experiment are described in Table 1. These were the Center for Climate System Research Ocean Component Model (COCO), a regional model developed at the University of Tokyo [*Hasumi*, 2006; *Watanabe and Hasumi*, 2009]; the Estimating the Circulation and Climate of the Ocean, Phase II (ECCO2) nested model, based on the Massachusetts Institute of Technology general circulation model (MIT‐gcm) [e.g., *Losch et al*., 2010]; the Institute of Computational Mathematics and Mathematical Geophysics (ICMMG) regional model, developed at the Siberian Branch of the Russian Academy of Sciences [*Golubeva and Platov*, 2007]; a regional North Atlantic/Arctic Ocean Sea Ice Model (NAOSIM), developed at the Alfred Wegener Institute for Polar and Marine Research and derived from the Geophysical Fluid Dynamics Laboratory Modular Ocean Model MOM‐2 [*Pacanowski*, 1995]; the Ocean Circulation and Climate Advanced Model (OCCAM) global sea ice‐ocean model developed at the National Oceanography Centre [e.g., *Aksenov et al*., 2010, 2011] and based on the Geophysical Fluid Dynamics Laboratory MOM; and the ORCA global sea ice‐ocean model of the National Oceanography Centre Southampton, developed within the Nucleus for European Modeling of the Ocean (NEMO) framework for ocean climate research and operational oceanography (http://www.nemo-ocean.eu/) [*Madec et al*., 1998].

All the six models participated in the previous phases 2 and 3 of the AOMIP experiments and were described in AOMIP publications [*Holloway et al*., 2007; *Proshutinsky et al*., 2011; *Jahn et al*., 2012; *Johnson et al*., 2012; *Clement Kinney et al*., 2014]; these publications and also other studies [e.g., *Köberle and Gerdes*, 2007; *Karcher et al*., 2003; *Kauker et al*., 2003; *Aksenov et al*., 2010, 2011] provided an analysis of the models' performance. In this section, only key aspects of model setup are given; additional model details relevant to the present study can be found in Table 1 and Appendix A. Details of all AOMIP models and their configurations also can be found at http://www.whoi.edu/projects/AOMIP.

The model horizontal resolution in the Arctic Ocean in the five models, COCO, ECCO2, ICMMG, NAOSIM, and ORCA, was similar, from 23 to 37 km, rendering these models as noneddying in the Arctic, whereas OCCAM was eddy permitting at the ∼8 km resolution [e.g., *Nurser and Bacon*, 2014]. In addition, two higher‐resolution ORCA 1/4° (eddy admitting) and 1/12° (eddy permitting/resolving) global configurations were used in the set of experiments to test sensitivity of the upper ocean dynamics to model resolution. The horizontal resolution of these configurations in the Arctic Ocean was ∼10 and ∼3 km, respectively.

Five models of the current model set (COCO, ICMMG, NAOSIM, OCCAM, and ORCA) used NCEP atmospheric reanalyses and one model (ECCO2) used JRA25 atmospheric forcing. For the present experiments, models were integrated over 1989–2007 from 41 year long spin‐ups for ICMMG, NAOSIM, and ORCA, a 20 year long spin‐up of COCO, a 4 year long spin‐up of OCCAM, and no spin‐up for ECCO2. The latter was forced with an optimized atmospheric forcing and lateral boundary fluxes, reducing model drift [*Nguyen et al*., 2011] (see next section and Table 1 and Appendix A for details). For the other models, the spin‐up was sufficiently long to spin‐up barotropic circulation and reached a state with a moderate baroclinic drift in the regional models. Global OCCAM clearly did not reach stabilization of the baroclinic circulation in the Arctic [e.g., *Aksenov et al*., 2010, 2011]. Global ORCA reached a state of a moderate ∼2% a^−1^ drift in the baroclinic circulation after ∼20 years (Q. Wang et al. [2015], personal communication).

Two out of the six models were global (OCCAM and ORCA), three were regional (COCO, ICMMG, and NAOSIM), and one (ECCO2) was a model nested in an outer global model with optimized oceanic exchanges through the open lateral boundaries at 55°N in the Atlantic and Pacific oceans. In two regional models, ICMMG and NAOSIM, the annual mean barotropic inflow in Bering Strait was constrained to its mean annual climatological value of 0.8 Sv [*Woodgate et al*., 2012; *Clement Kinney et al*., 2014] with the baroclinic component of the flow in the strait simulated in the models. In regional COCO, seasonal variability of 0.3 Sv is superimposed on the annual mean inflow of 0.8 Sv. In the other three models (ECCO2, OCCAM, and ORCA), the flow through Bering Strait was unconstrained.

Therefore, although the present study was not set up as a controlled forcing experiment, the wind forcing across the entire model set except one model (ECCO2) was the same. Given that the four models (COCO, ICMMG, OCCAM, and ORCA) used the same elastic‐viscous‐plastic (hereafter EVP) rheology in sea ice dynamical model [*Hunke and Dukowicz*, 1997], and the other two used Hibler's viscous‐plastic (hereafter VP) rheology [*Hibler*, 1979], the dynamical surface forcing of the ocean was similar in all models.

### 2.2 Experiments

To examine the pathways of PW in the Arctic Ocean in the models, a “passive tracer,” which does not affect water density and thus is passive with respect to ocean dynamics, was released in Bering Strait. The idea was to run models for the period 1989–2007 with an initial spin‐up for a 41 year from a state with the ocean and sea ice at rest (spin‐up period was shorter for some models) and to investigate PW tracer evolution, in particular area coverage and concentration, under the influences of external forcing. The challenge for this study was to identify periods that may represent a stable ocean circulation and link simulated PW tracer distribution patterns to external factors, such as PW inflow through Bering Strait and winds.

Under the assumption that wind forcing is the major factor responsible for ice and ocean circulation in the Arctic Ocean, we have used the AO index to identify periods of more or less stable atmospheric and oceanic circulation regimes. The AO index represents a dominant mode of the atmospheric sea level pressure (SLP) variability in the Northern Hemisphere [*Thompson and Wallace*, 1998]. The positive phase of the AO (1989–1996) [cf. *Steele et al*., 2004, Figure 11] corresponds to a lower‐than‐normal pressure over the polar region, which generates cyclonic (counter‐clockwise) winds over the Arctic Ocean, whereas the negative and near zero “neutral” phases of these indices (observed from 1997 to present) correspond to high pressure over the central Arctic Ocean, resulting in anticyclonic (clockwise) winds (Figure 1). We note that these periods of cyclonic and anticyclonic atmospheric forcing match very well with the behavior of the Arctic Ocean Oscillation index [*Proshutinsky and Johnson*, 1997; *Proshutinsky et al*., 2015].

The analysis of model results was focused on ocean circulation from 1989 to 1996 (high AO and a cyclonic atmospheric circulation) and from 1997 to 2007 (neutral AO and an anticyclonic circulation) [*Steele et al*., 2004].

Following the approach described above a set of three experiments was designed.

**Figure 2:**
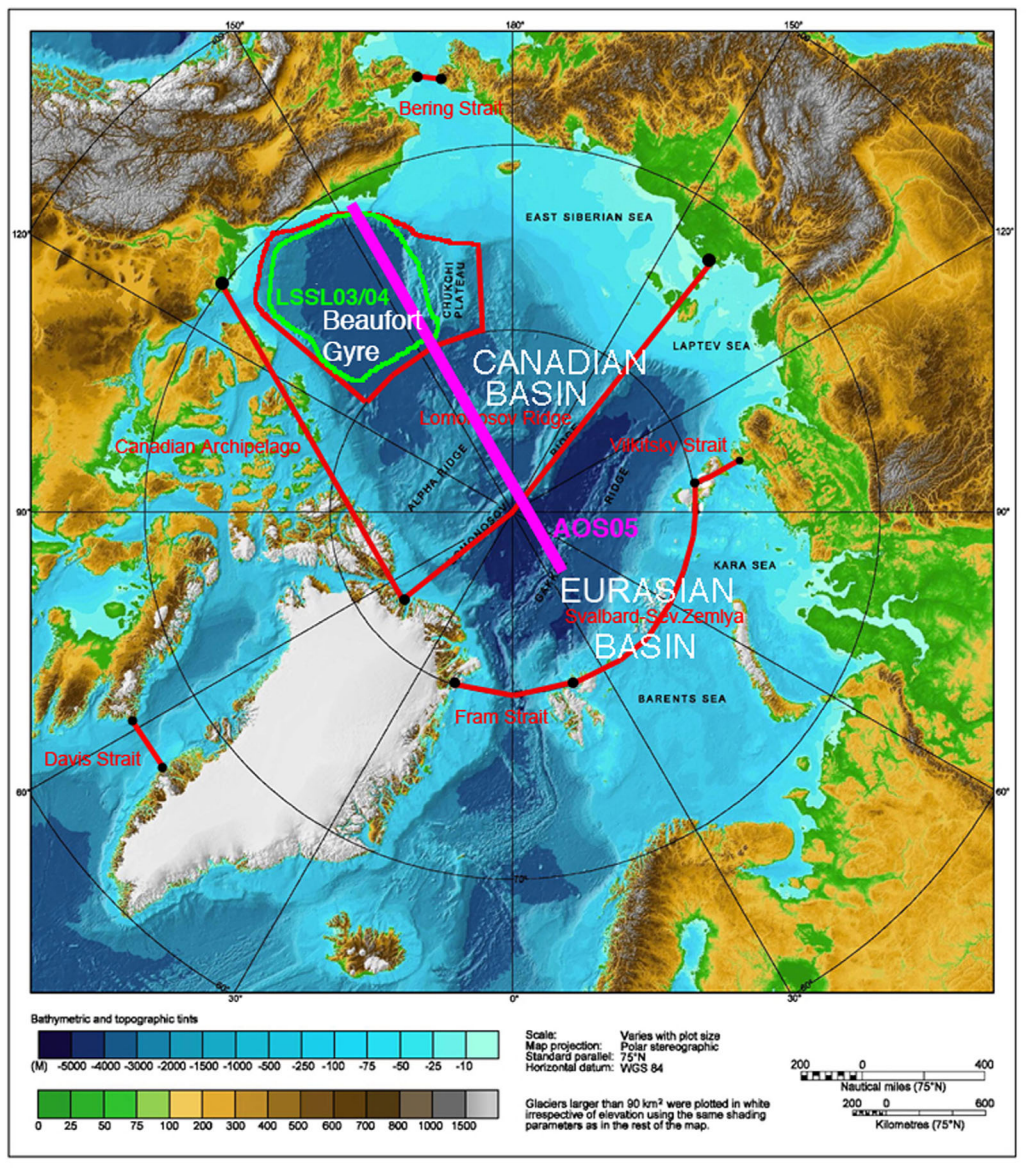
Schematic of the Arctic regions examined in the study over the IBCAO topography [*Jakobsson et al.*, 2012]. Boundaries of the Canadian Basin and Eurasian Basin of the Arctic Ocean and the Beaufort Gyre are shown with red lines. Sections across the principal Arctic straits, Fram Strait, Davis Strait, Vilkitsky Strait, and Bering Strait, used for the oceanic transports calculations are marked. Approximate position of the Beringia 2005 July–August cross Arctic observational transect (marked as AOS05) [*Jones et al*., 2008b] is shown by the magenta line. Area occupied by the CCGS *Louis S. St‐Laurent* summer cruises in 2003 and 2004 (denoted as LSSL03/04) [*Yamamoto‐Kawai et al*., 2008] is marked with the green line.

*Experiment 1*. In the first coordinated experiment (hereinafter Exp1), at the beginning of the period 1989–1996 (i.e., on the 1st January 1989, 0:00 hours GMT) the passive PW tracer was set to unity through the whole water column at a section across Bering Strait, and to zero elsewhere (Figure 2). Throughout the integration, the tracer was maintained at unity on the Bering Strait section but was allowed to evolve with full model dynamics in the rest of the model domain. At the start of 1997 the PW tracer was reset to zero everywhere, and then the tracer was set again to unity on the 1st January 1997 (0:00 hours GMT) in Bering Strait and was allowed to evolve again over the period 1997–2007 in the same manner as for the period 1989–1996. The experiment was performed with all six models. The aim of this experiment was to investigate changes in PW circulation under different wind forcing.

*Experiment 2*. During the second model experiment (hereinafter Exp2), the tracer was released in Bering Strait as in Exp1 but throughout the period 1958–2007, starting on the 1 January 1958, on 0:00 hours GMT, in order to investigate possible drivers of PW variability and the PW contribution to Arctic fresh water. The experiment was performed with three models—ICMMG, NAOSIM, and ORCA.

*Experiment 3*. This experiment (hereinafter Exp3) was designed to test the sensitivity of the simulated ocean circulation to the horizontal resolution and atmospheric forcing and was performed only with the ORCA 1°, 1/4°, and 1/12° global configurations, since there were three existing runs at different resolutions with the same model configuration. In the experiment, the passive PW tracer was released in Bering Strait as in Exp1 but throughout the period 1983–2007 in the ORCA 1° configuration. The model stream functions of the ocean circulation, oceanic relative vorticity, and Ekman pumping for the cyclonic 1989–1996 and anticyclonic 1997–2007 periods were analyzed to examine forcing mechanisms for the PW dynamics and pathways.

### 2.3 Model Diagnostics

For the analysis presented here, monthly and annual volume, heat and freshwater oceanic transports between the Arctic Ocean and Pacific and Atlantic oceans were calculated at key sections across Bering, Fram and Davis Straits and the straits of the Canadian Arctic Archipelago (Figure 2).

Monthly time series of PW content (PWC) and freshwater content (FWC) were obtained for each of the Arctic regions depicted in Figure 2. Unless stated otherwise, the FWC is calculated for each model grid‐cell relative to the reference salinity *S_ref_* = 34.8, which is the estimated mean salinity of the Arctic [*Aagaard and Carmack*, 1989]. Only positive FWC is taken into account in calculating FWC of the selected region over the part of the water column where the salinity is less than or equal to the reference salinity (see equation (B1), Appendix B). The simulated PWC was diagnosed by integrating the Pacific tracer fraction in each model grid‐cell over the water depth and over each region (equation (B2), Appendix B). In addition, two‐dimensional monthly and annual fields of vertically integrated PW fraction were calculated to examine PW inventory in the Arctic Ocean. This field was expressed in meters and represented the equivalent PW thickness in the water column [Y*amamoto‐Kawai et al*., 2008]. A similar diagnostic was computed for the liquid FW inventory [*Rabe et al*., 2011, 2014; *Jahn et al*., 2012].

To compare model results with the observations by *Jones and Anderson* [2008] and Jones et al. [2008a, 2008b] in the Arctic Ocean, Canadian Arctic Archipelago and in Fram Strait, the contribution of Pacific Water to the total fresh water (PFW) has been defined from PW fraction and the PW mean salinity, taken as *S_pw_* = 32.0 [*Jones et al*., 2008a] following equation (B3) (Appendix B). This is a simplification, since the salinity of PW varies significantly throughout the year [Woodgate et al., 2005a]. In these calculations, the reference salinity *S_ref_* = 34.8 is taken, so PW is the product of PW fraction and a constant scaling factor (1 − *S_pw_*/*S_ref_*). We also derived the vertically integrated content of the Pacific fraction of fresh water (PFWC) (Appendix B). Finally, the Ekman pumping is calculated for the Beaufort Gyre and Canadian Basin (Figure 2) from the curl of the combined stress, equal to the sum of wind stress on the open ocean surface, weighted by open water fraction, and stress at the sea ice‐ocean interface for sea ice‐covered areas, weighted by sea ice fraction; hereafter we refer to this diagnostic as the curl of total surface stress (see equations (B4a) and (B4b) in Appendix B).

The model validation relies on the available data from moorings and hydrographic transects. The time series of volume, heat, and freshwater fluxes through Bering Strait constructed from the long‐term in situ measurements from a site just north of the strait (mooring A3) [e.g., *Woodgate et al*., 2005a, 2005b, 2010] were used to evaluate model exchanges with the North Pacific. The model volume and freshwater transports through Fram and Davis Straits were compared with the observational estimates from moorings of *Schauer et al*. [2008] and *Curry et al*. [2011] .

Simulated PW parameters and PW fractions in the Arctic Ocean were compared with published observed distributions of these parameters obtained from various Arctic cruises: (a) the trans‐Arctic cruise of the Ice Breaker *Oden* in July–August 2005, Arctic Ocean Section 2005 (hereinafter AOS05) which began from the Alaskan shelf in the vicinity of Barrow and crossed the Arctic Ocean and ended near Svalbard [*Jones et al*., 2008b] and (b) the 2003 and 2004 observations collected during the CCGS *Louis S. St‐Laurent* summer cruises (hereinafter LSSL03/04) [Y*amamoto‐Kawai et al*., 2008]. For both observational data sets AOS05 and LSSL03/04 estimates of PW fraction were obtained by applying a four end‐member analysis (PW, meteoric water, sea‐ice melt, and Atlantic Water) to the temperature, salinity, dissolved inorganic nutrients, and dissolved oxygen measurements at the stations.

The vertical distribution of PW fraction across the Fram and Davis Straits estimated by *Jones et al.* [2008a] and *Jones and Anderson* [2008] from end‐member analysis was compared with the simulated fractions of PW tracer in these straits. However, these results need to be treated with a degree of caution, as simulated PW tracer distributions in the Arctic Ocean reached quasi‐equilibrium only in the models integrated for 1958–2007, whereas in the other models, these distributions were still in the transient state. Other observations [e.g., *Alkire et al*., 2010, 2007; *Carmack et al*., 2008; *Falck et al*., 2005] were used only for a qualitative comparison between measurements and model results, since a more comprehensive comparison was beyond the scope of this study.

## 3 Results of the Numerical Experiments

All six models listed in Table 1 participated in Exp1. The second experiment (Exp2) was carried out with the ICMMG, NAOSIM, and ORCA models, since only these three were integrated long enough (more than 10 years) to reach a quasi‐equilibrium state for the Pacific tracer in the Arctic Ocean. We focused on the dynamics of the Pacific layer in the Arctic Ocean in the models.

The models' performance was assessed by examining the inflow through Bering Strait and the outflows through Fram and Davis Straits, with a focus on the upper circulation in the Arctic Ocean. Once validated, we used the model results to identify PW pathways through the Arctic Ocean and diagnose the Arctic freshwater content change due to changes in the PW storage in different regions of the Arctic Ocean. We did not investigate different types of PW as defined by *Steele et al*. [2004] (i.e., summer and winter waters and Alaskan Coastal Water) because such an analysis required multiple tracers and was beyond the scope of the present study (examination of the pathways of different PW modes is given by, e.g., *Nguyen et al*. [2012] and *Watanabe* [2013]).

### 3.1 Simulated Transports Through Arctic Straits

In this section, only the comparison relevant to the present analysis is given, but more detailed assessments of each model may be found elsewhere [e.g., *Karcher et al*., 2005; *Golubeva and Platov*, 2007; *Holloway et al*., 2007; *Gerdes et al*., 2008; *Watanabe*, 2013; *Aksenov et al*., 2010; *Popova et al*., 2010; *Nguyen et al*., 2011; *Jahn et al*., 2012; *Johnson et al*., 2012; *Clement Kinney et al*., 2014]. Tables 2 and 3 present the comparison between simulated volume transports and oceanic (liquid) freshwater fluxes (relative to 34.8) through key Arctic straits and the best available observational estimates calculated for comparison for the same periods. ECCO2 and ORCA models showed consistency with observations in simulating Bering Strait inflow, while OCCAM had a lower inflow (0.5 Sv) due to the channel representing Bering Strait in the model being too shallow. The other models had the mean annual Bering Strait inflow constrained to its mean annual climatological value of 0.8 Sv, as detailed in section 2 (Table 1). A detailed analysis of the Bering Strait inflow is presented by *Clement Kinney et al*. [2014]. It will be shown here that despite the fact that Bering Strait inflow sets the PW source for the Chukchi and Beaufort Seas, the PW dynamics, pathways and, ultimately, PW outflows through Fram Strait and the straits of Canadian Arctic Archipelago primarily depend on the ocean dynamics, specifically the wind forcing, Ekman pumping, and changes in the ocean relative vorticity.

**Table 2:** Mean Net Volume Ocean Transports (Sv) through the Arctic Straits (Sections in Figure 2) Simulated in Six AOMIP Models and the Corresponding Observational Estimates^a^

Straits	ORCA1	ICMMG	NAOSIM	COCO	ECCO2^b^	OCCAM	Observations
Bering	**1.0** ± **0.5**	**0.8** c	**0.8** c	**0.8** ± **0.3**	**1.0** ± **0.3**	0.5 ± 0.2	**0.8** ± **0.2** d
Fram	**−2.2** ± **1.4**	**−1.7** ± **0.5**	**−2.9** ± **1.6**	**−2.2** ± **1.1**	**−0.2** ± **0.5**	**−1.1** ± **1.0**	**−1.8** ± **5.0** e
Davis	**−1.7** ± **0.5**	−0.5 ± 0.4	−0.6 ± 0.4	−0.7 ± 0.4	**−1.6** ± **0.4**	**−2.0** ± **0.3**	**−2.3** ± **1.0** f

a Model transports are averaged for the same periods as the observed (see keys), except for ECCO2 which was integrated from 1992 onward.

b ECCO2 transports are for 1992–2007.

c Prescribed barotropic inflow in the Arctic Ocean.

d 1991–2007 average from moorings in Bering Strait, may underestimate the volume transport by <20% [*Woodgate et al*., 2010, 2012].

e 1997–2006 average [*Fieg et al*., 2010].

f 1987–1990 averages including West Greenland shelf circulation [*Cuny et al*., 2005; *Curry et al*., 2011]. Model transports within the error bars of the observational estimates and the corresponding observational estimates are shown in bold.

**Table 3:** Mean Net Freshwater Ocean Transports (mSv) through the Arctic Straits (Sections in Figure 2) Simulated in Six AOMIP Models and the Corresponding Observational Estimates^a^. All Transports Are Referenced to the Salinity of 34.8

Straits	ORCA1	ICMMG	NAOSIM	COCO	ECCO2^b^	OCCAM	Observations
Bering	**80** ± **39**	**75** ± **12**	39 ± 7	**82** ± **25**	**85** ± **29**	36 ± 11	**79** ± **10** c
Fram	**−44** ± **18**	−224 ± 52	**−85** ± **26**	**−37** ± **26**	**−49** ± **23**	**−55** ± **12**	**−66** ± **18** d
Davis	**−95** ± **26**	−15 ± 32	−20 ± 14	−40 ± 15	**−80** ± **27**	**−86** ± **14**	**−92** ± **34** e

a Model transports are averaged for the same periods as the observed (see keys), except for ECCO2, which was integrated from 1992 onward.

b ECCO2 transports are for 1992 onward.

c 1990–2004 average [*Woodgate and Aagaard*, 2005].

d 1998–2008 average, including East Greenland Shelf [*de Steur et al*., 2009].

e 1987–1990 average including West Greenland shelf circulation [*Curry et al*., 2011]. Model transports within the error bars of the observational estimates and the corresponding observational estimates are shown in bold.

The main difference between the simulations appears in the partitioning of the Arctic outflow between the straits of the Canadian Arctic Archipelago and Fram Strait: ECCO2 and OCCAM simulate stronger outflow through the Canadian Straits and weaker outflow through Fram Strait, whereas for COCO, ICMMG, NAOSIM, and ORCA, the opposite is evident (Table 2). In Exp3, volume transports in ORCA 1/12° through Davis and Fram Straits were 1.9 ± 0.8 and 1.8 ± 0.8 Sv, within 11% of ORCA 1°. In NAOSIM with 1/4° and 1/12° resolution regional configurations, the transports through Fram Strait differ by less than 10% [*Fieg et al*., 2010]. In spite of the different partitioning of the outflow, all models had total liquid freshwater export from the Arctic Ocean with a range for the total freshwater flux through Davis and Fram Straits of 105–239 mSv (1 mSv = 10^−3^ Sv = 10^3^ m^3^ s^−1^). These values are close to the observational estimates of 159 ± 34 mSv (Table 2). Caution should be exercised in interpreting the observational estimates of the flow in Davis and Fram Straits. The flux estimates not only vary seasonally and interannually, which is reflected in the error estimates in Table 2, but also depend on the positions of the instruments on the transect and the methods of calculating ocean fluxes (detailed discussions are given for example by *Melling et al*. [2008], *Schauer et al*. [2008], and *Curry et al*. [2011]).

### 3.2 Pacific Water Distribution

**Figure 3:**
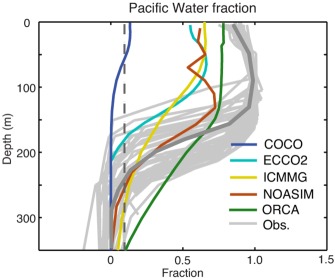
Averaged 2003–2004 summer (July–August) vertical profiles of Pacific Water (PW) fraction in the Beaufort Sea simulated in the AOMIP models (color) and from observations (light grey shows individual stations and dark grey shows mean profile) [*Yamamoto‐Kawai et al*., 2008]. Dashed line marks 0.1 of PW fraction. The area of averaging in the models coincides with the area of the measurements (Figure 2).

The mean summer 2003/2004 vertical distributions of the PW fraction in the Beaufort Sea and Central Arctic Ocean simulated in Exp1 were compared with the observations from the LSSL03/04 and AOS05 cruises in the same area (observational transects are shown schematically in Figure 2). The LSSL03/04 observations provide evidence of a high (>∼0.8) PW fraction within the top ∼200 m of the water column, sharply decreasing below this depth (individual station profiles in Figure 3). These results are consistent with other observations [e.g., *Carmack et al*., 2008] in the Canada Basin. From the observations, the layer of PW does not extend below the upper ∼250 m (the base of the layer is defined as the depth of the 10% PW fraction, shown as a dashed grey line in Figure 3). All models in this study generally simulate a smaller PW fraction in the Beaufort Sea than the observations show (Figure 3). In ICMMG and NAOSIM, PW depth is in good correspondence with the data, and in particular the PW depth in NAOSIM agrees better with observations than in the other models, although the overall PW fraction in both these models is lower than observed (Figure 3). In the upper ∼200 m, ORCA agrees better with observations than the other models, while the ICMMG and NAOSIM show lower PW fractions than those observed in the top ∼150–200 m, but better agreement below this depth (Figure 3). In COCO and ECCO2 models, the simulated depth of the base of the PW layer is shallower (∼70 and ∼170 m, respectively) than in the observations (∼250 m). In COCO, the top ∼70 m of the ocean contains most of the PW; this model simulates a shallower depth of PW than the other models. The COCO and ECCO2 models simulate lower PW tracer fractions, probably due to the period of PW tracer release (1997–2007) being too short: the time series of PWC (not shown) in the Beaufort Sea from these models demonstrate that the volume of PW tracer in the Beaufort Sea is still increasing at the rate of ∼6000 km^3^ yr^−1^ at the end of the integration period.

From the observations, the typical age of the waters in the central Arctic Ocean at depths of 0–250 m, i.e., the depth range where PW resides, is up to 20 years [e.g., *Karcher et al*., 2010], suggesting that ∼20 year tracer release periods are needed for PWC to reach quasi‐equilibrium in this region. It should be noted that the barotropic circulation in the Arctic Ocean, diagnosed in terms of the kinetic energy of the barotropic velocity (KE), spins up in the models within months, whereas the baroclinic circulation requires a spin‐up of 5 years. Analysis of a longer model spun‐up is beyond the scope of the present study; however, in the Coordinated Ocean‐ice Reference Experiments Phase 2 (CORE‐2), the drift in the Arctic FWC settles within ∼240 years (drift is within 10%) in the CORE‐2 ensemble runs (Q. Wang et al. [2015], personal communication), and settles in ORCA within ∼120 years. The drift is driven by changes in the global ocean FW and stratification. FW in regional models with imposed lateral boundary conditions settles faster, within 20 years, with the PW distribution settling in 20 years.

**Figure 4:**
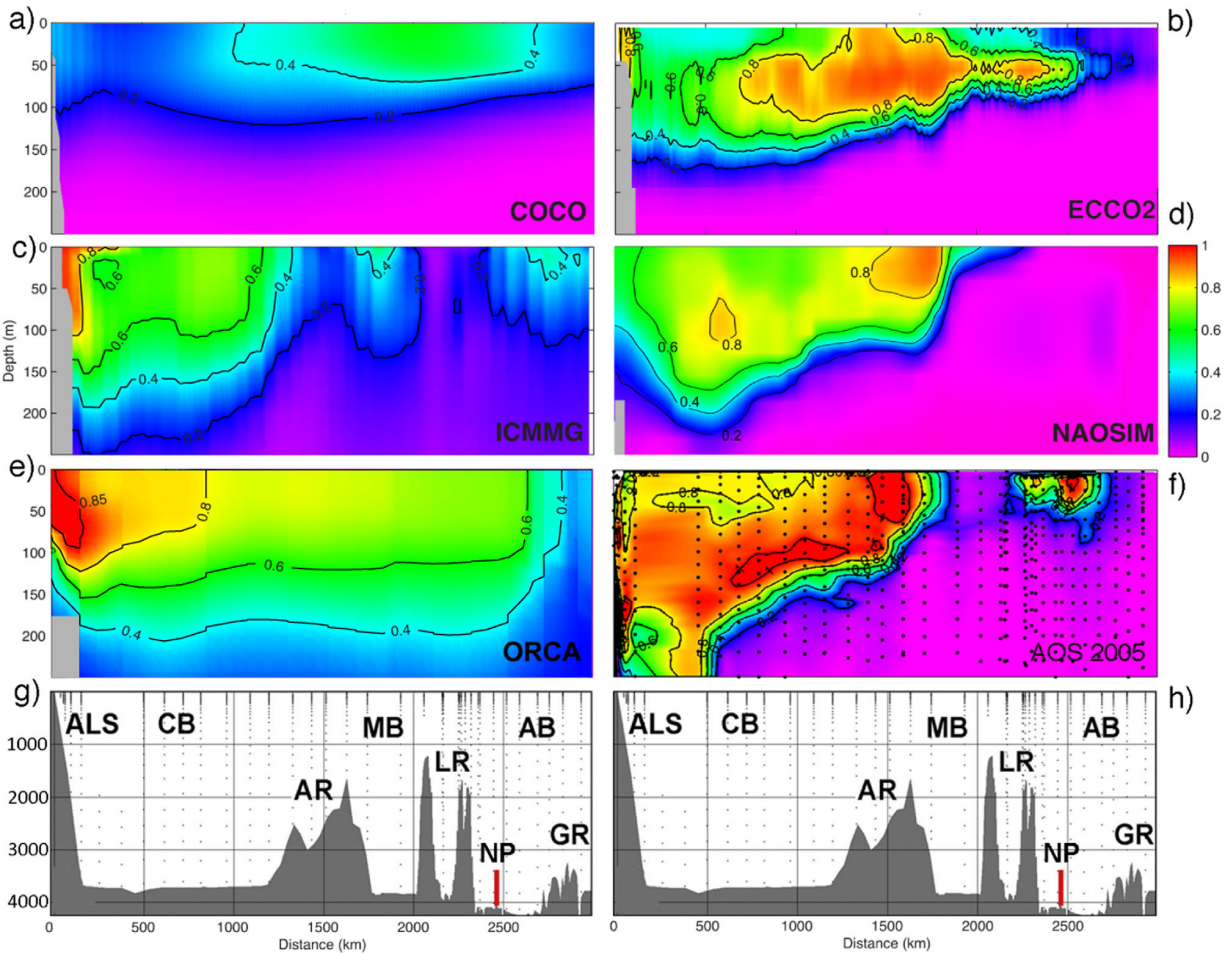
Simulated Pacific Water fraction in August–September 2005 across the central Arctic Ocean in (a) COCO, (b) ECCO2, (c) ICMMG, (d) NAOSIM, and (e) ORCA models. The model sections are chosen in the proximity of the track of the icebreaker *Oden* during the AOS05 cruise in Summer 2005 between the Alaskan shelf and Gakkel Ridge. (f) The PW fraction from the cruise data is shown together with the bathymetry along the ship track (g, h). The dots mark positions of the casts. Abbreviations: ALS, the Alaskan Shelf; AR, the Alpha Ridge; LR, the Lomonosov Ridge; GR, the Gakkel Ridge; NP, the North Pole; CB, Canada Basin; MB, the Makarov Basin; and AB, the Amundsen Basin.

The models suggest that the main volume of PW in 2005 was contained in the Canadian Basin with smaller fractions present in the Amundsen Basin (Figure 4); this is consistent with the summer 2005 observations from the AOS05 cruise. Figure 4f shows the PW fraction on the cross Arctic transect in 2005 calculated from the Redfield relations [*Jones et al*., 2008, 2008b] and the other plots show the simulated PW tracer on the same transect. The results clearly indicate that the PW distribution in the Canadian Basin differs between the models. In COCO, the principal part of the PW is present in the Makarov Basin and above the Lomonosov Ridge (Figure 4a). In ECCO2, ICMMG, and NAOSIM, the PW distribution is wedge shaped across the basin, deepening from ∼100 m in the Makarov Basin to ∼200–250 m near the Alaskan shelf (0.2‐isoline in Figures 4b–4d). In ORCA (Figure 4e) and OCCAM (not shown), the main fraction of PW is near the Alaskan shelf slope and in the southern Beaufort Sea.

Among the models analyzed here, the cross‐Arctic distributions of PW in ECCO2 and NAOSIM demonstrate the most similarity with the observations in simulating the wedge‐shaped distribution of PW (Figures 4b, 4d, and 4f). Both the ECCO2 and NAOSIM models simulate the observed high PW fraction above the Mendeleev‐Alpha Ridge at 50–70 m depth and the deepening of the layer of high PW fraction in the Canada Basin with a lower PW fraction in the upper ∼50–70 m in the Beaufort Sea (Figures 4b and 4d). In the Canada Basin, the ECCO2 and NAOSIM models show values about 0.1–0.2 too low (cf. Figures 4b, 4d, and 4f). ICMMG and ORCA simulate higher than observed PW fraction in the Canada Basin, and lower than observed PW fraction above the Mendeleev‐Alpha Ridge and in the Makarov Basin (Figures 4c, 4e, and 4f). In the OCCAM model, most of the PW is in the Canada Basin near the Alaskan shelf (not shown).

All the models in the study, except COCO, simulate an elevated (>0.6) fraction of PW tracer above the Alaskan shelf slope at depths ∼50–80 m (Figure 4). This coincides with the observed location of the Alaskan shelf‐break jet [e.g., *von Appen and Pickart*, 2012]. Similar to the observations, ECCO2 and NAOSIM show elevated PW tracer fractions above the Mendeleev‐Alpha Ridge in the upper ∼100 m (Figures 4b, 4d, and 4f). ECCO2 and ICMMG also simulate an increase in the PW fraction in the vicinity of the Lomonosov Ridge (cf. Figures 4b, 4c, and 4f). This is the result of PW flow along Arctic mid‐ocean ridges.

**Figure 5:**
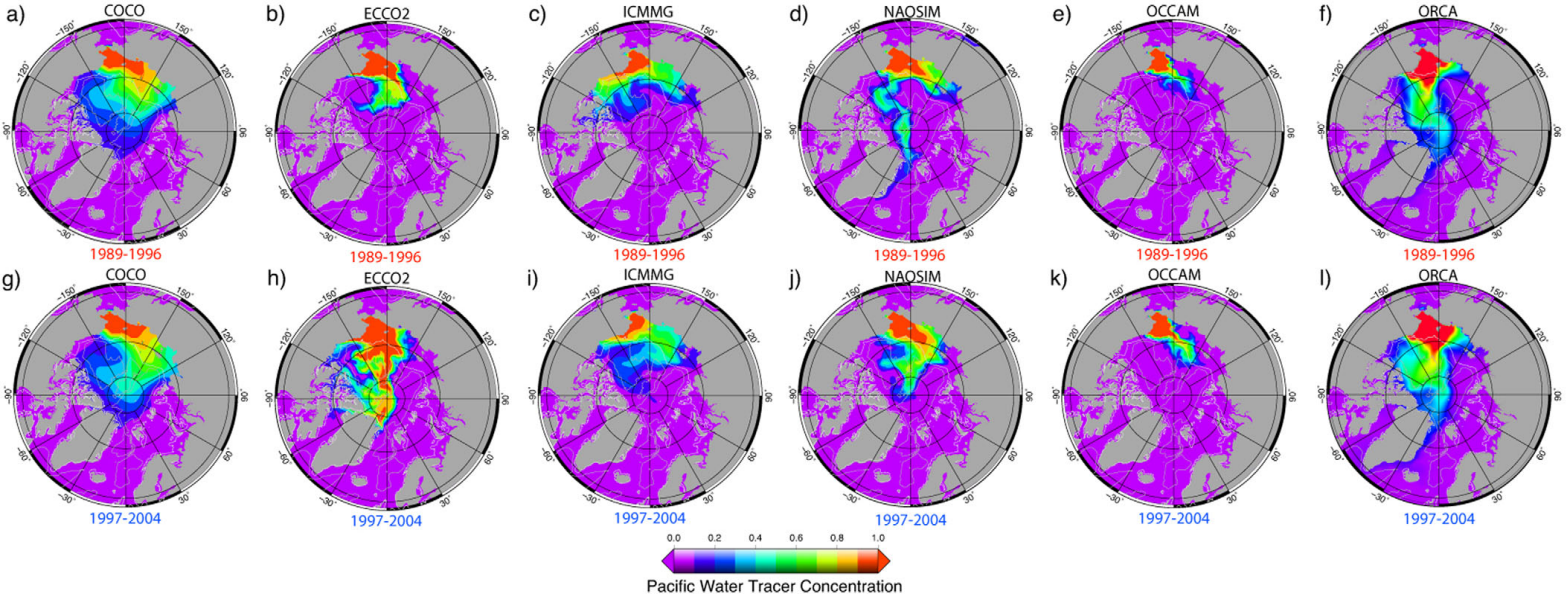
Annual mean fraction of the Pacific Water (PW) tracer (color) at ∼10 m depth simulated in the six AOMIP models: (a, g) COCO, (b, h) ECCO2, (c, i) ICMMG, (d, j) NAOSIM, (e, k) OCCAM, and (f, l) ORCA in the last year of the (a–f) cyclonic (high AO) 1989–1996 and (g–l) anticyclonic (low and neutral AO) 1997–2004 circulation periods. Model results are from Experiment 1 with the tracer initialized at the beginning of each circulation period.

**Figure 6:**
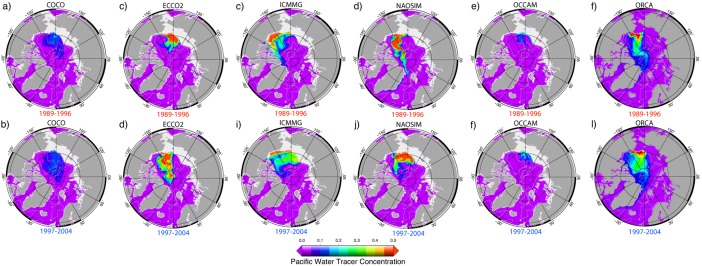
Same as Figure 5 but at ∼100 m depth.

During 1997–2004 in ICCMG, PW at the surface, as well as at ∼100 m, initially follows the Mendeleev‐Alpha Ridge, then turns toward the Beaufort Sea, with part of this flow branching toward Fram Strait (Figures 5i and 6i). In NAOSIM, PW at the surface flows along and above Siberian shelf and then across the Arctic Ocean along the Lomonosov Ridge toward Fram Strait, whereas at the depths of ∼100 m, PW flows along the Mendeleev‐Alpha Ridge in the model (Figures 5j and 6j). In ECCO2, PW spreads across the Arctic Ocean from the Siberian shelf toward the Canadian Arctic Archipelago and Fram Strait along the Mendeleev‐Alpha Ridge as a uniform flow between the surface and ∼150 m (Figure 6d).

Summarizing, all models participating in the analysis, except NAOSIM, simulate a shallower than observed PW layer in the Canada Basin. ECCO2 and NAOSIM show the best agreement of the PW distribution across the Arctic Ocean with the AOS05 measurements. On the other hand, ORCA demonstrates the best agreement between the observed during the LSSL03/04 cruise and simulated spatial mean PW fraction in the Beaufort Sea in the top ∼200 m in 2003/2004, although the simulated PW fraction is too low in this model. In all models, the total simulated PW content is lower than observed, probably due to the relatively short periods of the PW tracer releases in Exp1 (∼10 years). One of the implications of this analysis is that the short‐period PW model tracer releases can be used for pathways examination but have a limited application in the analysis of the PW content in the Arctic Ocean.

### 3.3 Pacific Water Pathways

For the 1989–1996 cyclonic, and 1997–2007 anticyclonic circulation regimes, the models simulate two pathways of PW in the Arctic Ocean (Figures 5 and 6). The eastern pathway first follows the Alaskan coast, then crosses the Beaufort Sea and leaves the Arctic Ocean through the straits of the Canadian Arctic Archipelago and through the western part of Fram Strait. In the simulations, PW is present in western Baffin Bay, the western Davis Strait and in the East Greenland Current (Figures 5 and 6). PW following the western route, flows into the western Chukchi Sea and the East Siberian Sea, then crosses the Arctic Ocean along the Mendeleev‐Alpha Ridge or along the Lomonosov Ridge within the Transpolar Drift current and, finally, exits the Arctic through the Canadian Arctic Archipelago and western part of Fram Strait. The models generally show a lateral spread of the PW tracer away from the shelf slope in the East Siberian and Chukchi Sea into the Arctic interior, which is consistent with observations [e.g., *Timmermans et al*., 2014].

The shelf route enables PW to spread westward toward the Siberian shelf. If there were no wind, under the influence of the Pacific‐Atlantic ocean sea level pressure gradient, PW would flow into the Arctic and then turn east along the Alaskan Shelf slope due to the Coriolis force [e.g., *Timmermans et al*., 2014]. Wind forces PW water to go west along the Siberian Shelf. The alternation between the western and eastern PW route is reflected in the changes in the position of the front between PW and Atlantic‐derived waters.

The models show several differences in simulating PW pathways. In the COCO and OCCAM models, PW mainly follows the western pathway (Figure 6), whereas in ORCA, it follows the eastern pathway (Figure 5). In ECCO2, PW mostly follows the western pathway with a portion of PW entrained in the current along the Alaskan shelf (Figure 6). In the NAOSIM model, the PW pathways alternate between the eastern and western routes depending on the atmospheric circulation (Figure 5). This is in agreement with the conclusions of *Steele et al*. [2004]. The pattern of the PW spread in ICMMG, NAOSIM, and ORCA during the cyclonic period 1989–1996 exhibits a strong trans‐Arctic flow from the East Siberian Sea into Fram Strait, whereas during the anticyclonic period 1997–2007, the PW outflow is reduced (Figure 6). The other models do not show such an obvious relationship between the spread of the Pacific tracer in Exp1 and the type of atmospheric circulation.

In all models but COCO, which exhibits a sharp decrease of PW fraction with depth, the spread of PW is similar in the top 100 m (Figure 6). In the deeper layers (100–250 m, not shown), PW is entrained in the cyclonic circulation, but at this depth, the PW fraction is small and does not contribute substantially to the PW content in the Arctic Ocean.

**Figure 7:**
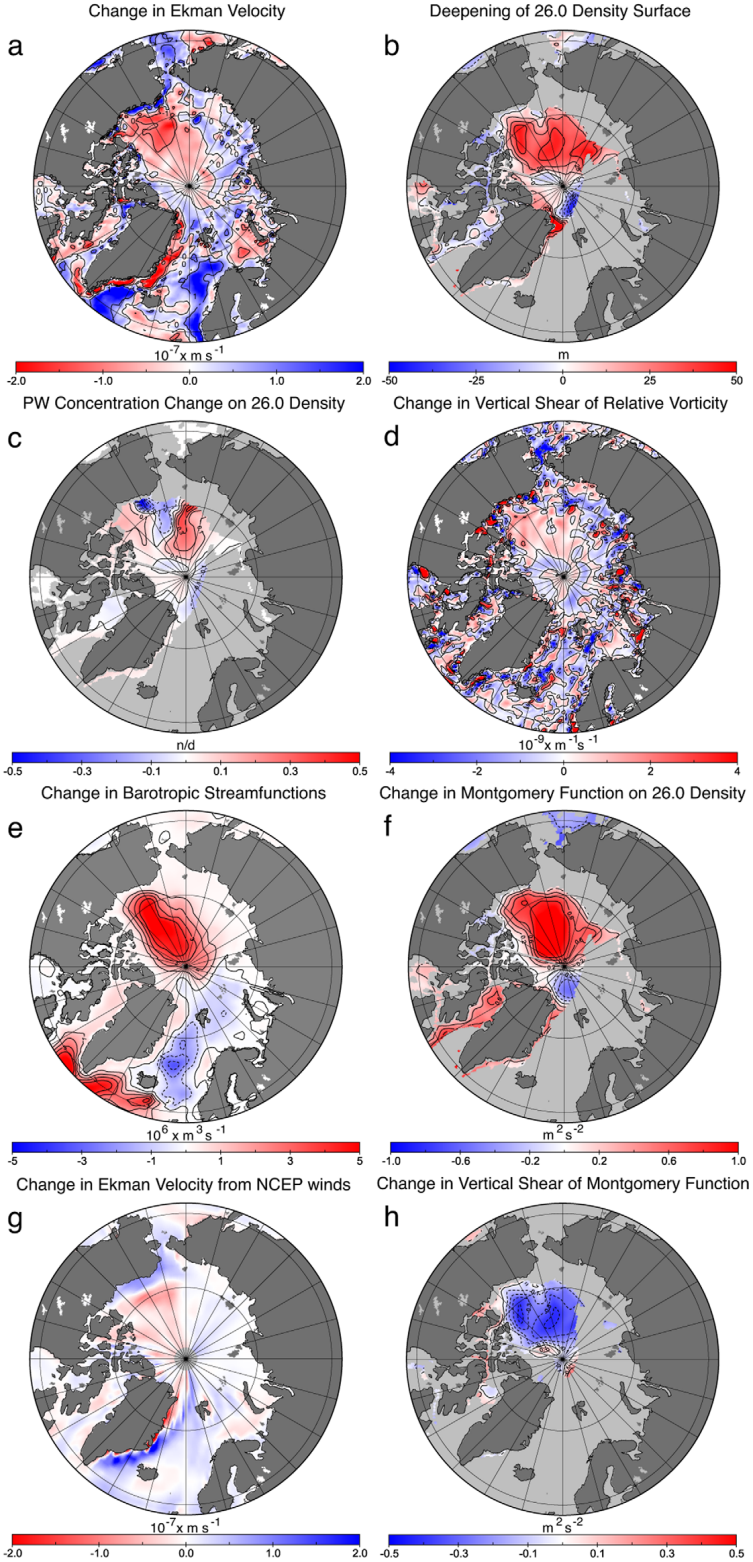
Simulated change between the cyclonic (1989–1996) and anticyclonic (1997–2004) AO circulation periods in ORCA. (a) The Ekman vertical velocity (Ekman pumping) change between cyclonic and anti‐cyclonic periods; (b) the deepening of the 26.0 density surface; (c) the PW concentration change on the same density surface; (d) the change in the vertical shear of relative vorticity between the surface and 100 m depth; (e) the change in the oceanic barotropic streamfunctions; (f) the change in the Montgomery function on the 26.0 density surface; (g) the change in the Ekman vertical velocity calculated from the NCEP winds; (h) the change in the vertical shear of the Montgomery function between the 25.5 and 26.5 density surfaces. Results are from Experiment 3 where the tracer was initialized at the beginning of each circulation period.

**Figure 8:**
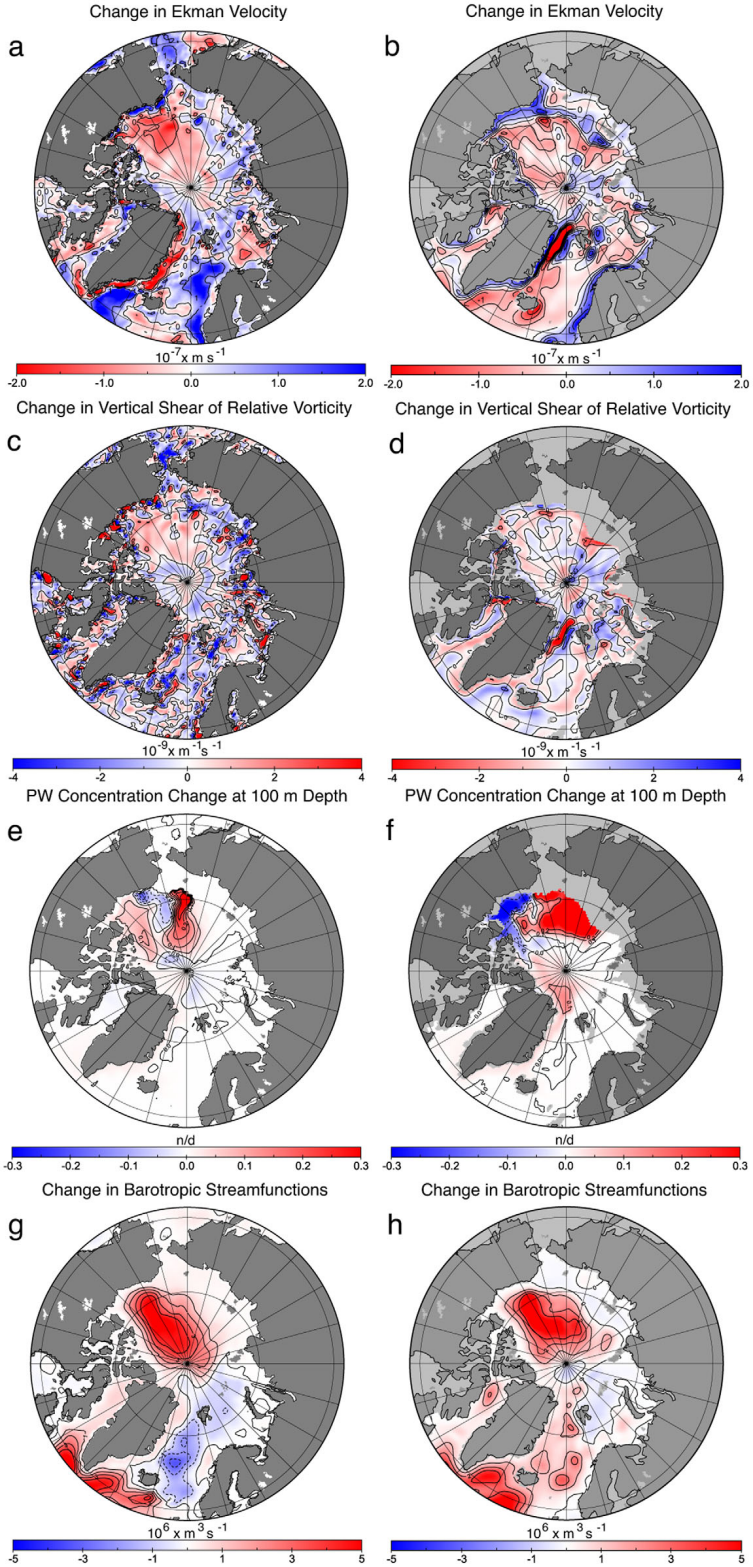
Simulated changes between the cyclonic (1989–1996) and anticyclonic (1997–2004) AO circulation periods in (a, c, e, g) ORCA and (b, d, f, h) ICMMG. (a, b) The Ekman vertical velocity change; (c, d) the change in the vertical shear of relative vorticity between the surface and 100 m depth; (e, f) the change in the PW concentration at 100 m depth; (g, h) the change in the oceanic barotropic streamfunctions. Results are from Experiment 3 run where the tracer was initialized at the beginning of each circulation period.

The simulations show consistency between increased vertical downward Ekman velocity (pumping) between 1989–1996 and 1997–2004 and deepening of the pseudopotential density surfaces (25.5, 26.0, and 26.5) (Figures 7a and 7b). This led to the strengthening of the anticyclonic flow in the Canadian Basin, advecting more PW along the Lomonosov Ridge (Figure 7c). On the other hand, Ekman pumping increase and deepening of the PW layer resulted in steeper horizontal gradients in layer depth and stronger lateral density gradients, thus in a stronger vertical shear in horizontal velocity and therefore stronger vertical shear in relative vorticity (Figure 7d). Stronger anticyclonic relative vorticity is expressed partly as a baroclinic response of the circulation, i.e., stronger anticyclonic vorticity/circulation near the surface than below and partly as a barotropic response (Figure 7e). Finally, change of shear associated with Montgomery potential [*Aksenov et al*., 2011] (Figure 7f) is linked with increase in vertical vorticity shear. Two models, ICMMG and ORCA, showed similar change in the barotropic stream function, in Ekman pumping, relative vorticity at the surface and 100 m and PW concentration (Figure 8), suggesting that the Ekman driving mechanism is not model specific.

Following this line of argument, the following hypothesis can be formulated. The increase in the anticyclonicity of the winds in the Canada Basin in the second half of the 1990s resulted in a stronger Ekman downwelling in the center of the Beaufort Gyre, with a compensating upwelling along the gyre periphery (Figure 7b). This increased pressure in the center Beaufort Gyre and spun‐up anticyclonic subsurface (down to ∼100 m) flow around the gyre periphery (Figures 7e and 7f), redistributing PW into the East Siberian Sea and Makarov Basin (Figure 7b).

### 3.4 Changes in Pacific Water and Arctic Freshwater Content

**Figure 9:**
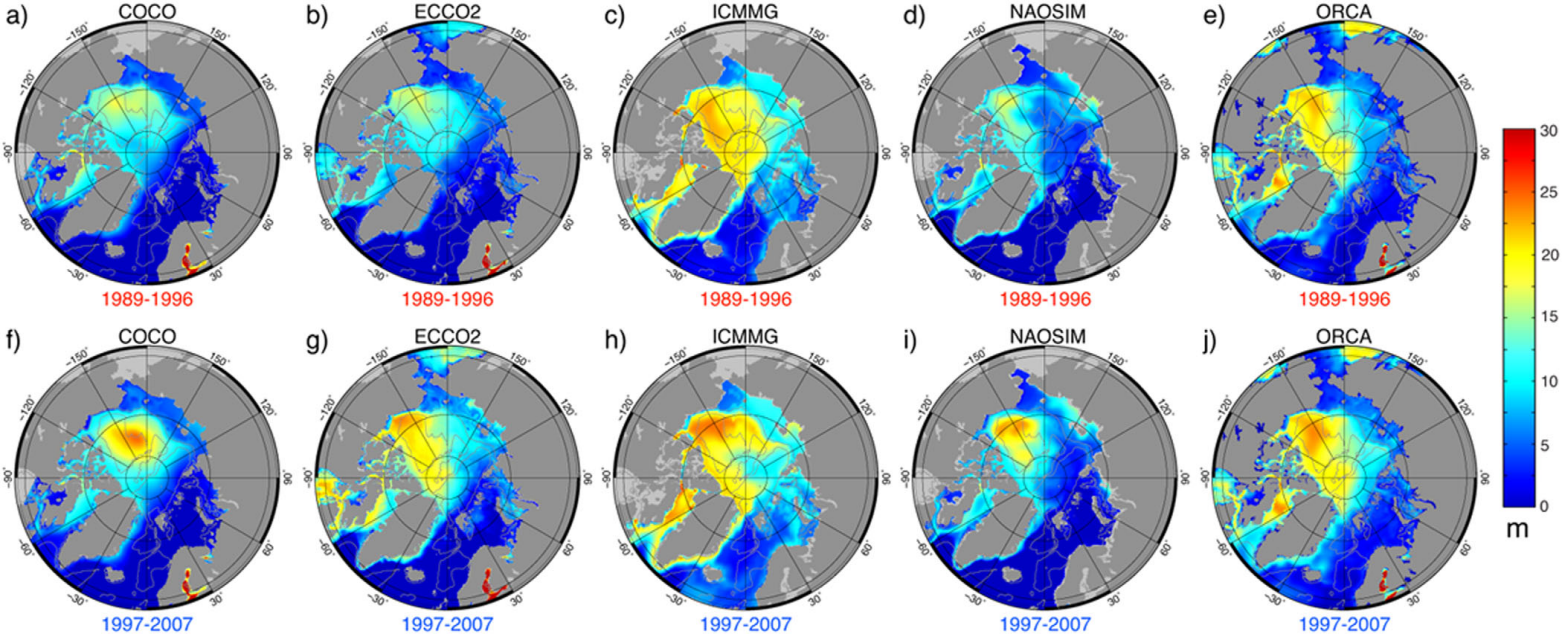
Annual mean freshwater content (FWC) in the top 500 m simulated in the five AOMIP models. FWC was calculated according to equation (B1) (Appendix B) for the last year of the cyclonic (a–e) 1989–1996 (high AO) and anticyclonic (f–j) 1997–2007 (low and neutral AO) circulation periods (a, f) COCO, (b, g) ECCO2, (c, h) ICMMG, (d, i) NAOSIM and (e, j) ORCA.

**Figure 10:**
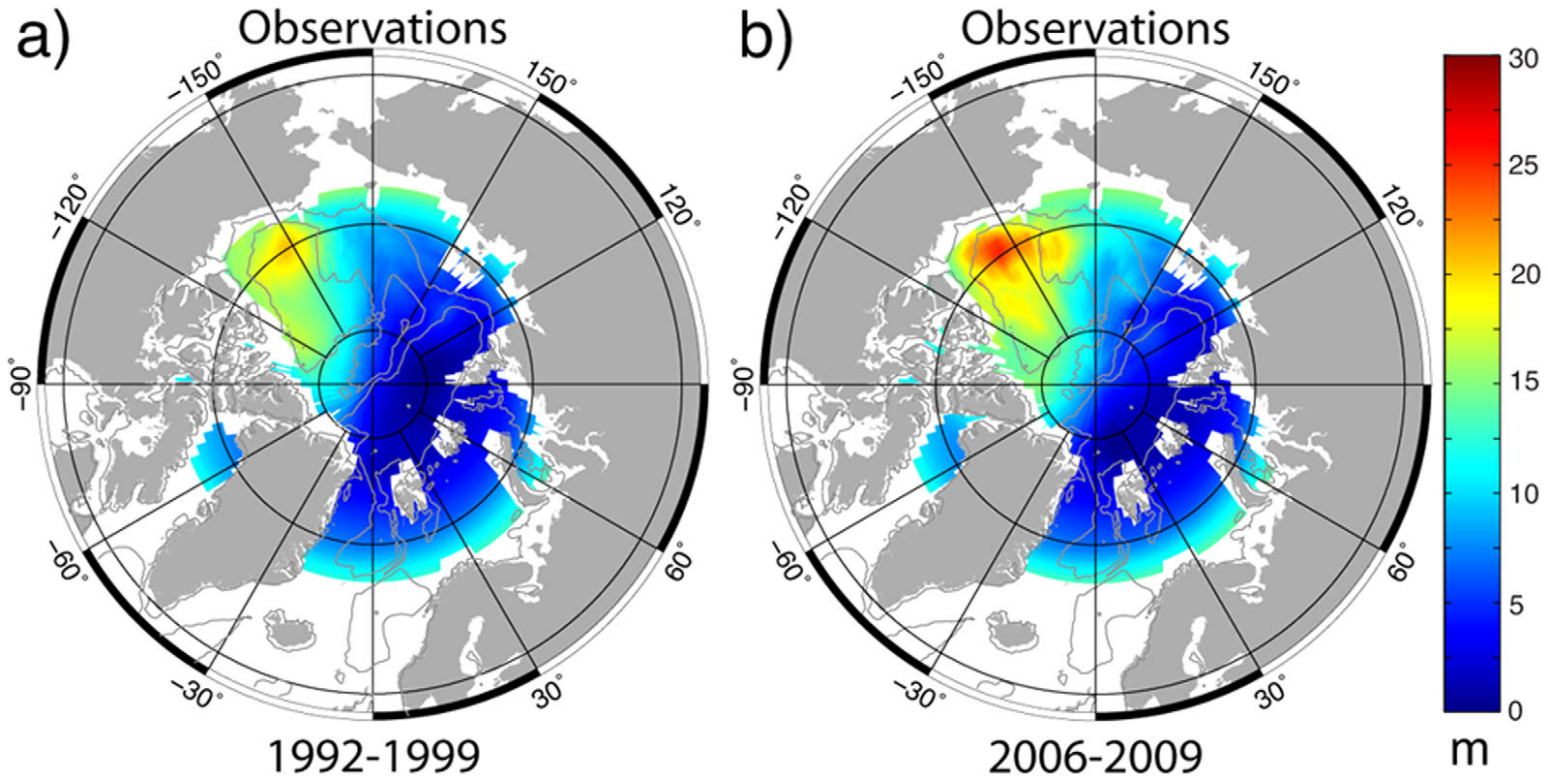
Climatology of freshwater content (FWC) in the Arctic Basin for 1992–1999 (a) and 2006–2009 (b) (shown in colors). Data courtesy Ben Rabe (Alfred Wegener Institute, Germany).

The spatial distribution of FWC (referenced to 34.8) in all six models shows maxima of 20–27 m in the Canadian Basin, decreasing toward the Eurasian Basin of the Arctic Ocean (Figure 9). This pattern is consistent with observations, e.g., from Arctic Climatology (Figure 10), *Rabe et al*. [2011, Figure 2], *Carmack et al*. [2008, Figure 7.7], and *Serreze et al*. [2006, Figure 4].

**Figure 11:**
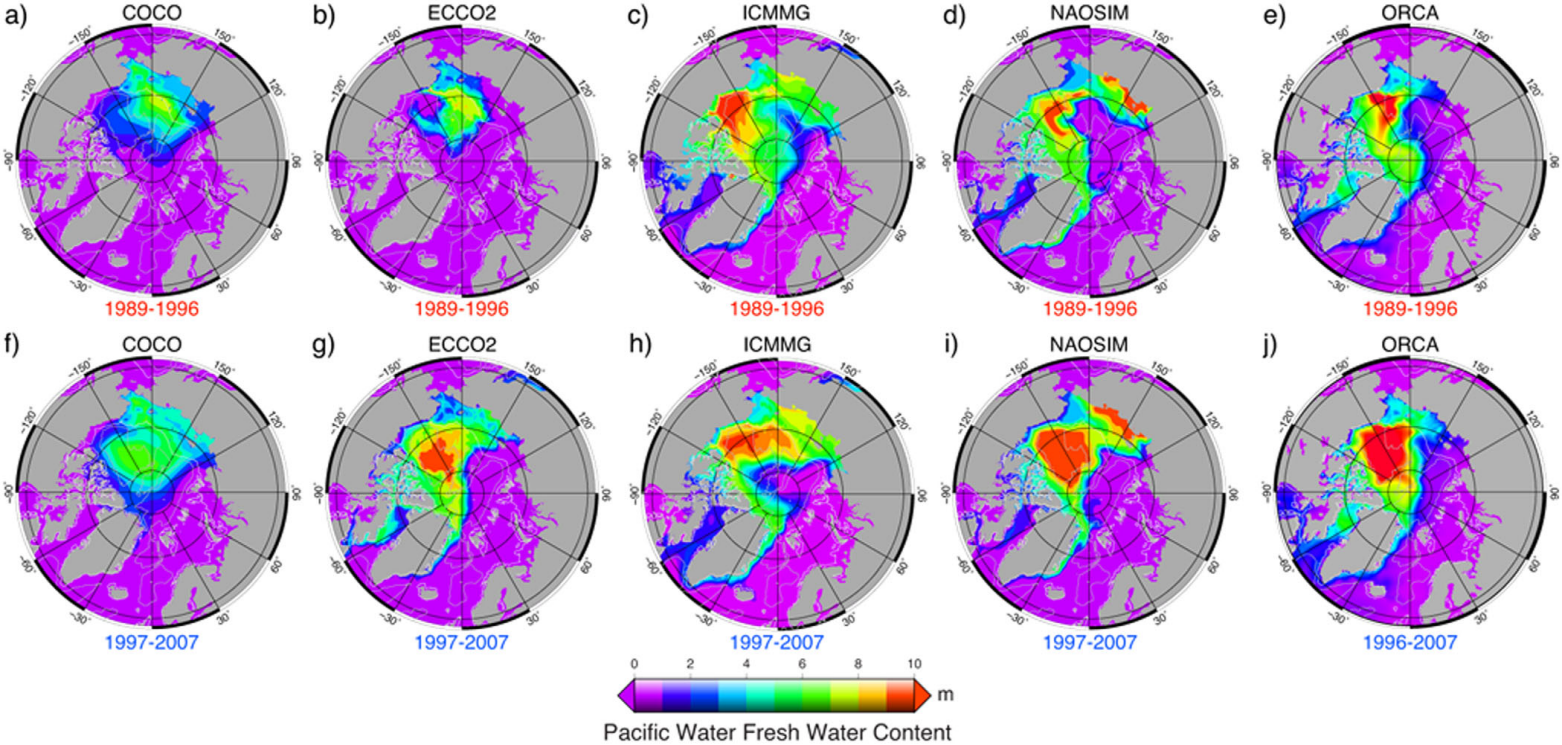
Annual mean freshwater content of the Pacific Water (PFWC) simulated in the five AOMIP models. PFWC was calculated according to equation (B3) (Appendix B) in the last year of the (a–e,) cyclonic 1989–1996 (high AO) and (f–j) anticyclonic 1997–2007 (low and neutral AO) circulation periods (a, f) COCO, (b, g) ECCO2, (c, h) ICMMG, (d, i) NAOSIM and (e, j) ORCA.

Although different in detail, the vertically integrated PFWC in the models exhibits a similar distribution with maxima in the Canadian Basin; a pattern which is also evident in the observations (cf. Figure 11 in this study with *Carmack et al*. [2008, Figure 7.12]). Pacific fresh water makes up a large fraction of the total fresh water in most of the Canadian Basin, whereas in the Eurasian Basin, the PFWC is small (Figure 11).

In runs of ECCO2, ICMMG, NAOSIM, and ORCA integrated with a multidecadal PW tracer release (Exp2), the PWC is ∼200 m in the Canadian Basin (not shown), similar to the observed value of ∼220 m [e.g., *Carmack et al*., 2008]. A sharp gradient in simulated PW fraction between these two basins marks the front separating Pacific and Atlantic Waters.

In the last decade, with a strong anticyclonic atmospheric circulation dominating the Arctic Ocean (a neutral AO state), the Pacific‐Atlantic front has partially recovered to its pre‐1990s position [e.g., *Alkire et al*., 2007; *Abrahamsen et al*., 2009; *Karcher et al*., 2012]. Overall, the model results (except ECCO2 and OCCAM) exhibit a similar variability at the surface with the eastward retreat of the front from the Makarov Basin during the cyclonic period 1989–1996, and the return of the front during the anticyclonic period 1997–2007 (Figure 5). All models simulate a substantial fraction of PW, advected from the Chukchi Sea by surface westward flow over the Siberian shelf via deLong Strait into the East Siberian Sea. PW further flows to the Laptev Seas via the straits of the New Siberian Islands (cf. Figures 5 and 6). High FWC in the models is evident in the Canadian Arctic Archipelago, where the PWC is also high, and in Baffin Bay, where PWC is generally low except for the Baffin Island Current on the western side of the bay.

In summary, long‐term variations in the vertically integrated total FW and PW distributions in the models reflect the cyclonic (high AO) and anticyclonic (lower or neutral AO) circulation regimes and show accumulation of the total and Pacific fresh water in the Beaufort Sea during the anticyclonic years and a decrease of their storage during cyclonic years (Figures 9 and 11).

**Figure 12:**
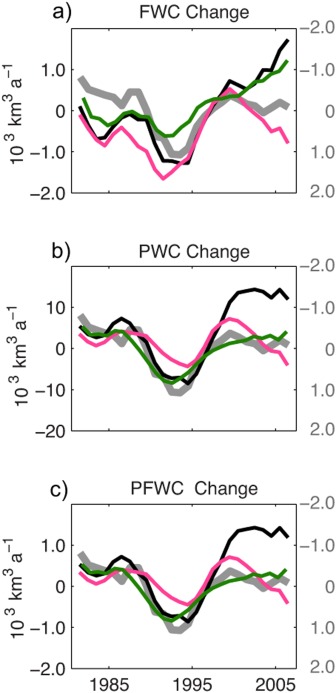
Five year running means of rate of change of (a) freshwater content referenced to *S_ref_* = 34.85, (b) Pacific Water content, and (c) Pacific freshwater content in the ORCA, ICMMG, and NAOSIM models in the Canadian Basin in km^3^/yr (vertical axes on the left), shown by the solid green, magenta, and black lines, respectively. The region is in Figure 2. Light grey lines show 5 year running mean of Arctic Oscillation index (AO) indices. The AO indices are scaled for easier comparison with other time series (vertical axes in gray on the right).

We use the ICMMG, NAOSIM, and ORCA model results for 1958–2007 to quantify the contribution of PW to the Arctic freshwater content variability in our simulations. We use results from continuous tracer releases for five decades (Exp2). Figure 12 shows time series of the 5 year mean of the total freshwater content change, PW and Pacific Water FWC calculated for the Canada and Makarov Basins of the Arctic Ocean (aka the Canadian Basin) (Figure 2). There is a decrease in the overall Arctic FWC in the 1980s and 1990s and a large increase in the freshwater storage from the late 1990s to 2007 (Figure 12). Most of the variability in total Arctic FWC is due to variations in the Canadian Basin, with at least half of this variability occurring in the Beaufort Gyre. The contribution of the Eurasian Arctic (the Nansen and Amundsen Basins and Laptev Sea) is smaller (not shown). On time scales of a few years, freshwater buildup alternates with periods of freshwater decrease, and these periods qualitatively appear to correspond to the cyclonic (high AO) and anticyclonic (low or neutral AO) periods of the Arctic oceanic and atmospheric circulation (grey lines in Figure 12). In fact, 5 year mean time series of FWC change in the Canadian Basin in all three models correlate with AO indices (0.7, 0.9, and 0.8 at 95% confidence level for ICMMG, NAOSIM, and ORCA). The most pronounced correlation of 0.9 (at 95% confidence level) between AO and FWC change in the NAOSIM simulations occurs in the late 1980s to 1990s, when the high AO indices and the strong cyclonic circulation regime coincided with a large ∼6000 km^3^ loss of Arctic freshwater content, and in the decade 1997–2007 with lower and neutral AO induces, when anticyclonic circulation was accompanied by a sharp increase in the freshwater storage (Figure 12). The models differ in the magnitude of the freshwater storage change. The NAOSIM has the largest freshwater storage change anomaly, with 1996–2007 peak‐to‐peak change in the upper Arctic Ocean of ∼7500 km^3^, in good agreement with that observed (∼8000 km^3^) [*Rabe et al*., 2011, 2014]. ORCA has a storage change of ∼7300 km^3^, whereas the ICMMG peak‐to‐peak change is ∼6000 km^3^.

PW that accumulated in the Canadian Basin prior to the 1980s was exported from the Arctic, such that the PW remaining in the basin was at a minimum in the late 1990s. Since the end of the 1990s, the PW content increased in the model simulations, surpassing the 1980s level by the mid‐2000s (Figure 12).

As in the FWC results above, the simulated interannual change of PWC is dominated by variations in the Canadian Basin. Correlations between PWC change in the Canadian Basin in 1981–2007 (this period was chosen to avoid drift of PWC at the beginning of the integrations) simulated in ICMMG, NAOSIM and ORCA and the AO are 0.7, 0.9 and 0.7, (the coefficients are calculated at 95% confidence level). Since the PFWC change is equal to the PWC change multiplied by a constant factor (Appendix B, equation (B3)), it is also highly correlated to the AO in these models (Figure 12).

At the interannual scale, correlations between FWC change and Ekman pumping (equations (B1), (B4a), and (B4b), Appendix B) in the Canadian Basin are 0.6 for ICMMG, 0.7 for NAOSIM, and 0.6 for ORCA at 95% confidence level. The negative curl of total surface stress generates convergence of Ekman transport and is responsible for Ekman pumping, resulting in freshwater accumulation. In the models, correlations between PWC change and Ekman pumping in this area are similar to those for FWC change (0.6 for ICMMG, 0.7 for NAOSIM, and 0.6 for ORCA at 95% confidence level).

From this analysis, we conclude that in the ISMMG, NAOSIM, and ORCA models, the interannual variations in the FWC and PWC accumulation in the Canadian Basin are mostly driven by wind. The strength of the Bering Strait inflow in ORCA shows a correlation of ∼0.5 at 95% confidence level with the change in the PW in the Canadian Basin, suggesting the inflow may be one of the sources of the variability. Note however that ICCMG and NAOSIM are regional models with constrained inflow through Bering Strait.

### 3.5 Seasonal Variations

Using a variety of hydrographic data, *Proshutinsky et al*. [2009] analyzed the seasonal cycle of liquid fresh water in the Beaufort Gyre and found it to correlate well with the seasonal cycle of wind stress curl over that region. They suggested that Ekman pumping is the main driving mechanism of liquid freshwater accumulation and release in the gyre. To test if the models simulate this seasonal correlation (including variations of the PW component) and to examine whether the strength of the correlation varies between periods of different types of circulation, we constructed time series of annual cycles of the FWC and PWC in the Canadian Basin and Beaufort Gyre from the monthly 1989–2007 time series. In the same manner, we calculated the seasonal cycle of curl of total surface stress, considering it as a proxy for Ekman pumping. Seasonal cycles were computed from the results of Exp2 carried out with the ICMMG, NAOSIM, and ORCA models for anticyclonic (1997–2007) and cyclonic (1989–1996) periods, as well as for the whole period 1989–2007. From these results, we calculated lag‐correlations between the curl of total surface stress and the rate of FWC increase. In these models, FWC in the Canadian Basin has a strong seasonal cycle with a maximum in August–October and a minimum in May–June. The simulated PWC also exhibits a strong seasonal cycle with the maximum volume change between spring and summer. Correlations between the curl of total surface stress and both the FWC and PWC, averaged in the Beaufort Gyre and the Canadian Basin, are negative, and typically high and significant, at −0.6 to −0.8 (95% confidence level). During anticyclonic years, the winter Ekman convergence due to negative wind curl (and also negative curl of total surface stress) is enhanced, resulting in greater accumulation of fresh water and PW in the Beaufort Gyre in winter, whereas during cyclonic years, it is reduced, resulting in reduced accumulation of the fresh water and spread of PW from the Beaufort Gyre into the rest of the Canadian Basin. In the models the seasonal time series of FWC appear to be correlated with total surface stress curl at 0.7–0.8 (95% confidence level) during the anticyclonic years 1997–2007, and uncorrelated during the cyclonic years 1989–1996. In contrast, the PWC is correlated with total surface stress curl at 0.6–0.8 (95% confidence level) through the whole period, with the higher correlation during the anticyclonic years 1997–2007. A similar relationship was found in the models for the Canadian Basin, except that the FWC seasonal time series is correlated with total surface stress curl at 0.6–0.7 (95% confidence level) during the cyclonic years 1989–1997. In the Canadian Basin, the curl of total surface stress leads the variation in the PW content by approximately 1 month, whereas in the Beaufort Gyre, leads by between zero and 4 months.

## 4 Discussion

### 4.1 Variability of Pacific Water in the Models

The model results show substantial variations in PW pathways and distribution between the models. Some models (i.e., NAOSIM in the present study) show accumulation of PW in the Canadian Basin during the anticyclonic 1997–2007 years and release of PW toward Fram Strait during the cyclonic 1989–1996 years. This is consistent with the accumulation of the surface fresh water due to negative curl of total surface stress over the Canadian Basin in anticyclonic years [e.g., *Proshutinsky et al*., 2002, 2009; *Rabe et al*., 2011; *Giles et al*., 2012]. Simulated integrated freshwater fields are approximately similar between models with a FWC maximum in the Canadian Basin, but model simulations of PW circulation differ significantly.

Since five out of six models in this study were forced with the same NCEP atmospheric winds, we examined whether the differences among the model circulations and PW pathways were related to different model resolutions, especially associated with how the models simulate topographically guided rim currents and ocean eddies. To test this, a set of sensitivity experiments (Exp3) were carried out with the ORCA model. These experiments included runs of the ORCA model with horizontal resolutions of 1/4°, 1°, and 1/12°.

**Figure 13:**
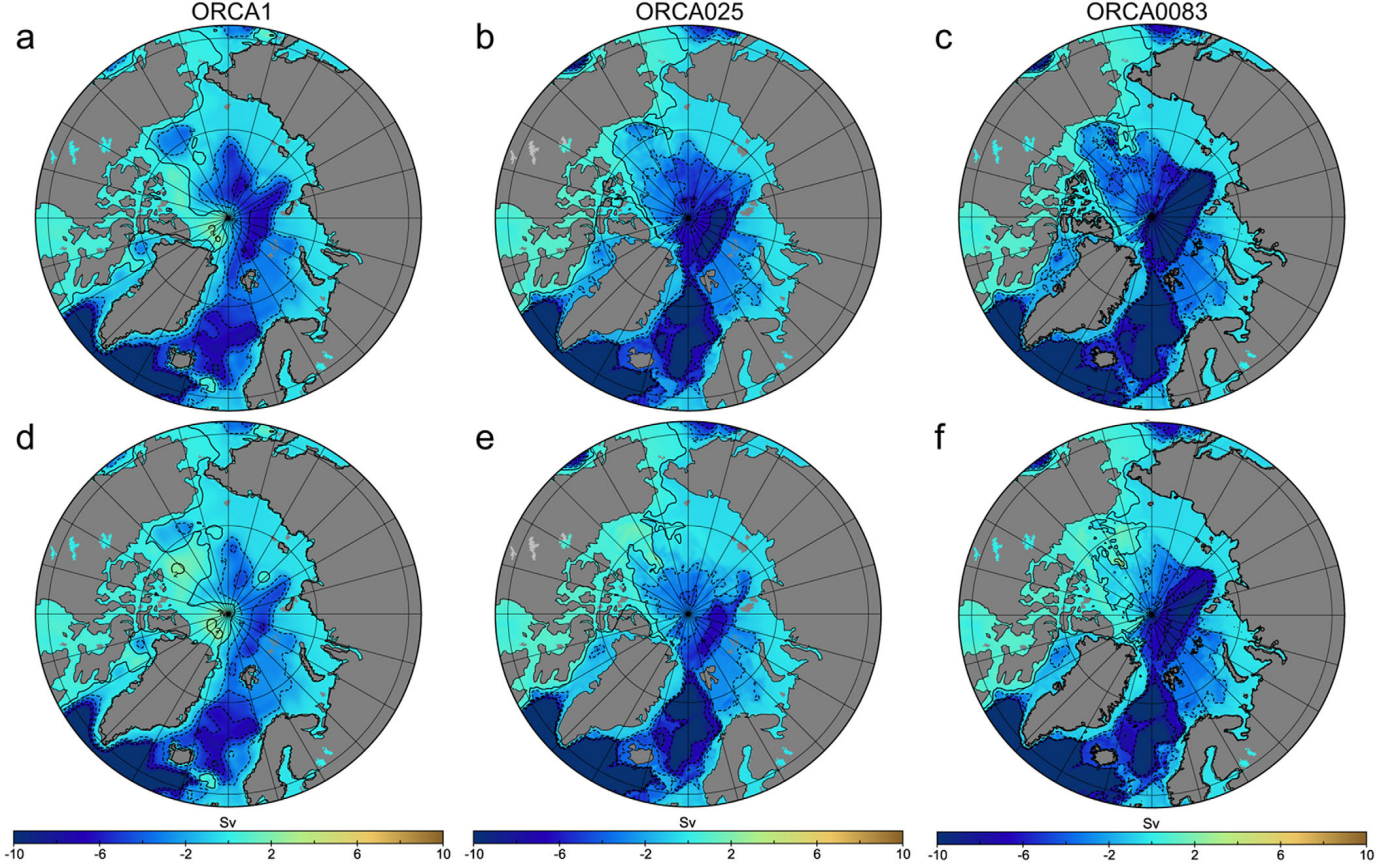
Simulated multiannual barotropic stream function Ψ (contours) in the (a–c) cyclonic (1989–1996) and (d–f) anticyclonic (1997–2004) AO circulation periods. Results are from the Experiment 3 run 1983–2007.

Increasing model resolution from 1° (not eddy resolving) to 1/4° (eddy admitting globally) and 1/12° (eddy permitting globally but eddy admitting in the Arctic at best) resulted in a more anticyclonic barotropic circulation in the Canadian Basin (Figure 13). *Holloway et al*. [2007] found that increasing model resolution from coarse to eddy permitting strengthens the cyclonic circulation in the Arctic Ocean due to the eddy rectification by topography. The change in model resolution also affected oceanic freshwater fluxes in and out of the Arctic Ocean, thus altering the stratification and ocean circulation. Exchanges through Fram Strait were only marginally (10%) stronger and flow through the Barents Sea was weaker in 1/4° and 1/12° than in the 1° model (cf. barotropic stream functions in Figure 13).

### 4.2 Mechanisms of Pacific Water Accumulation

From model results and observations [*Proshutinsky et al*., 2009], it appears that the negative curl of total surface stress generates convergence of Ekman transports and leads to Ekman pumping, acting as a primary driver of the seasonal variability of the FWC and PW in the Canadian Basin (correlation 0.7–0.8). There also appears to be a strong correspondence between the cyclonic and anticyclonic atmospheric circulation and the seasonal changes of PW characteristics studied in this paper. During anticyclonic years, the winter Ekman convergence due to negative wind stress curl (and also negative curl of total surface stress) is enhanced, whereas during cyclonic years, it is reduced. The high correlation between seasonal changes in the FWC, PWC and total surface stress curl in the Beaufort Gyre suggests that the principal mechanisms for these variations are the redistribution of the FWC and PW between the Beaufort Gyre and the rest of the Canadian Basin via accumulation or release caused by the wind.

On interannual time scales, FWC in the Beaufort Gyre and Canadian Basin simulated by NAOSIM shows a notable covariability with Ekman pumping [*Rabe et al*., 2011, Figure 6]. In the ICMMG, ORCA, and ORCA1 models, the correlation between FWC change and the Ekman pumping in the Canadian Basin was also high, in excess of 0.7 at the 95% confidence level. In contrast to FWC variability, in the ICMMG and NAOSIM models, the PWC change in the Canadian Basin demonstrated a higher correlation with the AO indices (coefficient up to 0.7–0.9 at 95% confidence level) than with local Ekman pumping (correlation coefficient 0.6–0.7 at 95% confidence level). Simulated PWC changes in the ORCA model were only weakly correlated with the AO, although the model shows a correlation of PWC variations with the Bering Strait inflow (∼0.5 at 95% confidence level).

### 4.3 Changes in the Pacific Water Export

**Figure 14:**
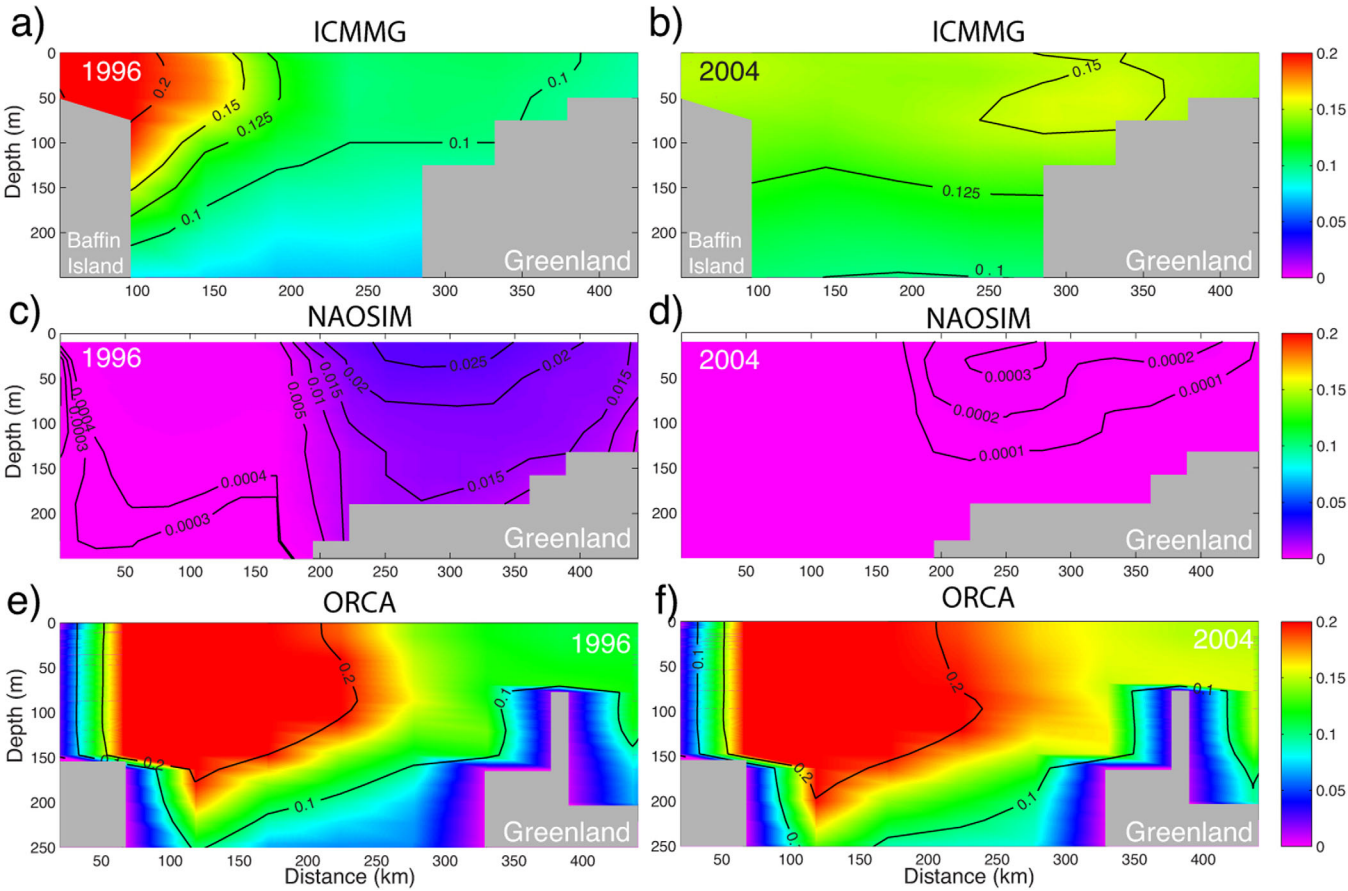
Annual mean 1996 (a, c, e) and 2004 (b, d, f) fractions of Pacific Water (PW) in Davis Strait in the (a‐c) ICMMG, (c‐f) NAOSIM, and (e, f) ORCA model. Experiment 2 with the continuous Pacific tracer release 1958–2007.

**Figure 15:**
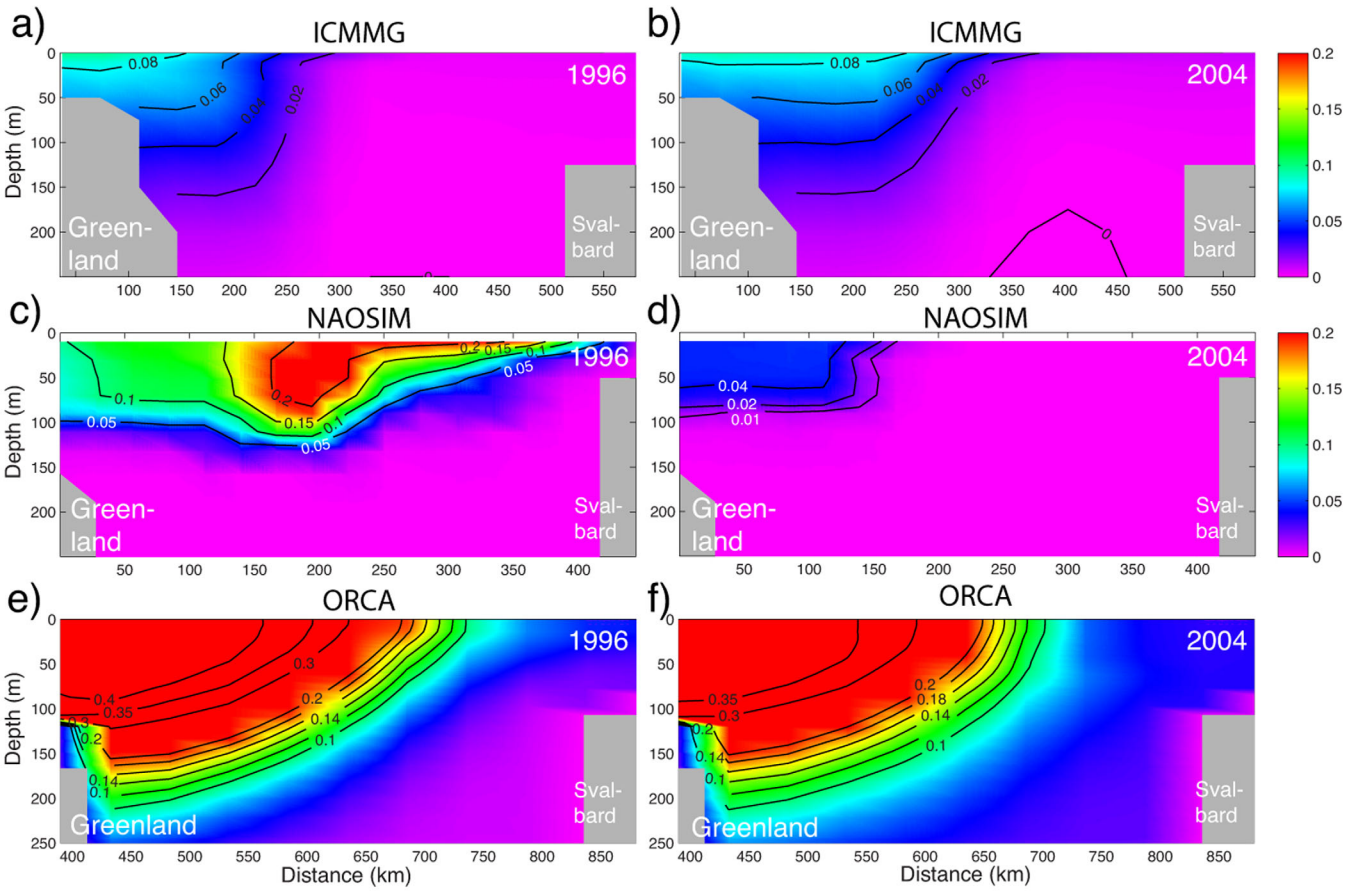
Same as Figure 14 but for Fram Strait.

**Figure 16:**
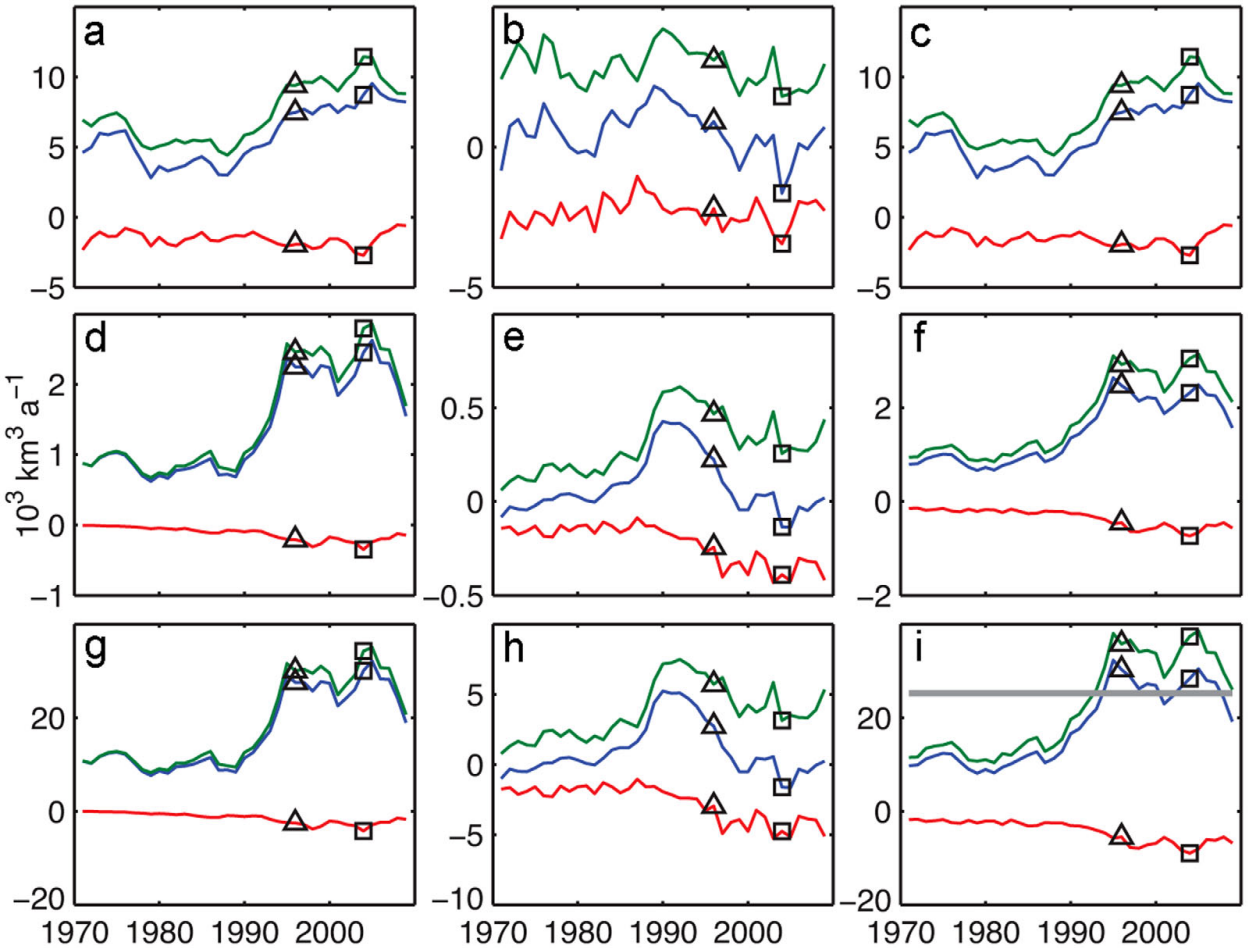
Time series of simulated annual mean (a–c) freshwater (FW), (d–f) Pacific freshwater (PFW), and (g–i) Pacific Water (PW) transports in (a, d, g) Fram Strait and (b, e, h) Davis Strait in the ICMMG model; sections are depicted in Figure 2. Blue, red, and green lines are net, northward (in the Arctic Ocean), and southward (out of the Arctic Ocean) transports, respectively. (c, f, i) Added transports through Fram and Davis Straits. Mean annual transports in 1996 and in 2004 are marked by triangles and squares. Grey line shows mean annual PW inflow through Bering Strait in the model (constant in ICMMG). Results are from the Experiment 2 with the continuous PW tracer release 1958–2007; only 1970–2007 period is shown.

**Figure 17:**
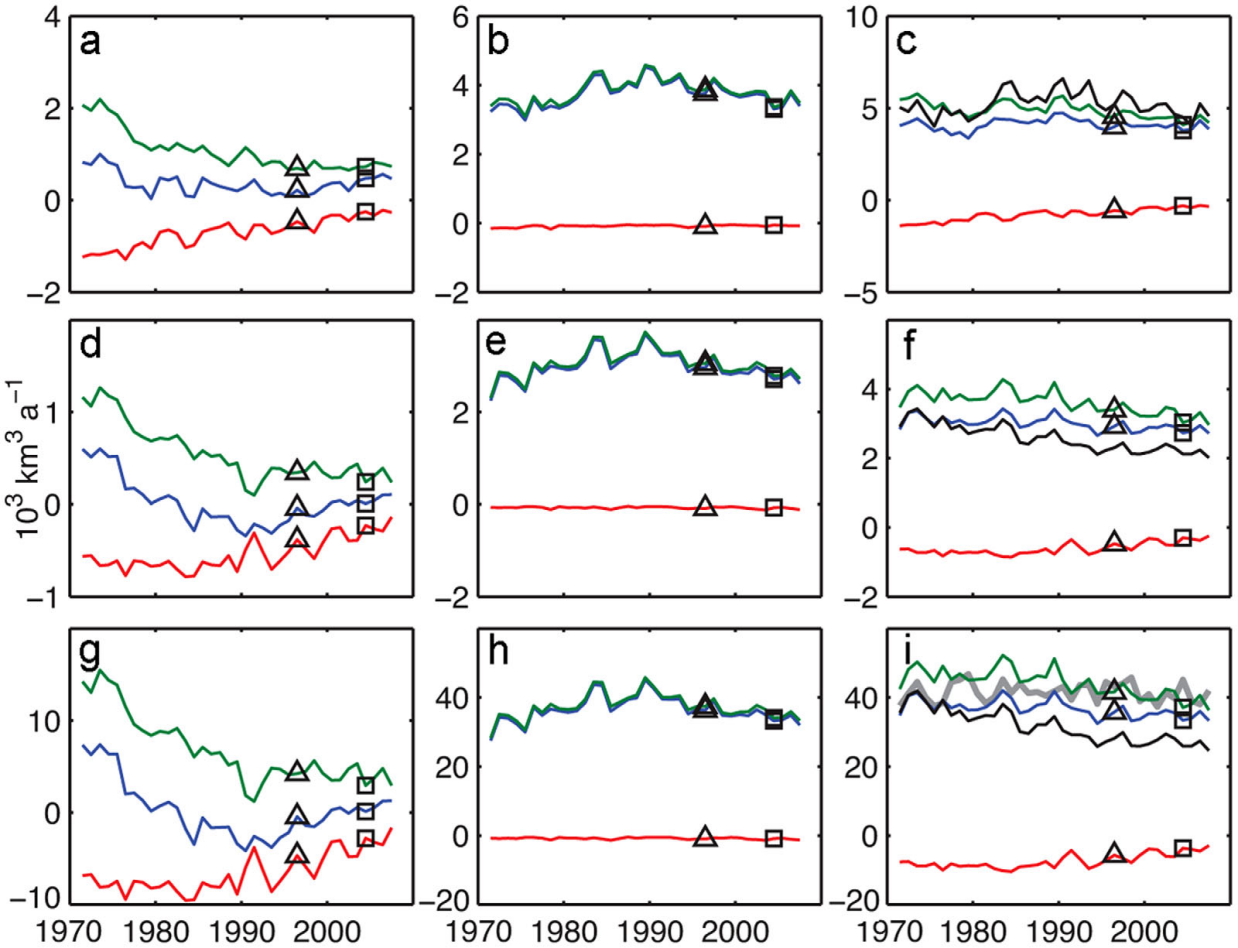
Same as Figure 16 but for ORCA model. Black lines in the right‐column are the total net transport of FW, PW, and PFW in the North Atlantic, obtained by adding transport in Fram and Davis Straits, across the Svalbard‐Severnaya Zemlya section and in Vilkitsky Strait (sections as in Figure 2).

**Figure 18:**
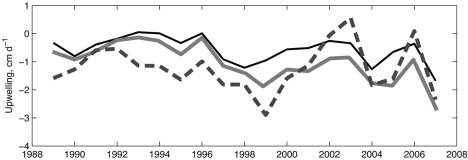
Ekman vertical velocity (pumping) calculated over the Canadian Basin (Figure 2) following equations (B4a) and (B4b) in Appendix B in ICMMG (black solid line), NAOSIM (black dashed line), and ORCA (solid light‐grey line) models. Positive velocity is upward (upwelling and Ekman suction) and negative velocity is downward (downwelling and Ekman pumping).

We now compare observed and simulated PW export in three models ICMMG, NAOSIM, and ORCA with the long‐term PW tracer release (Exp2) that reached quasi‐equilibrium of the PW tracer content in the Arctic Ocean. It should be noted that it takes ∼20 years for the models to reach quasi‐equilibrium between the inflow through Bering Strait and outflow of PW tracer from the Arctic. *Falck et al*. [2005] and *Dodd et al*. [2012] reported a decrease in the PW outflow through Fram Strait between 1997 and 2004 (followed by some increase in the PW outflow in 2008 and 2010–2011) [*Dodd et al*., 2012], which is believed to be due to the change in the Arctic circulation during anticyclonic years [e.g., *Steele et al*., 2004; *Karcher et al*., 2012]. The model results showed evidence of a similar trend in the Pacific outflow (Figures 14 and 15). The PW fraction in Fram Strait, although not vanishing completely, greatly reduced, but the total FW fraction declined only moderately (Figures 16 and 17). The latter is consistent with observations [e.g., *de Steur et al*., 2009; *Rabe et al*., 2011] and indicates the change in the source water for the outflow from the Arctic Ocean through the strait. In Davis Strait, in the ICMMG and ORCA models, there was a significant reduction of the PW content between 1996 and 2004, caused by the decrease of PW in the West Greenland Current (Figure 15). In contrast, the PW export through Davis Strait was unchanged, but the total freshwater content and its Pacific fraction export were reduced (Figure 16). In ICCMG and ORCA, both the total PW outflow from the Arctic and the combined transports through Fram and Davis Straits declined through the 1990s and 2000s (Figures 16 and 17). This is consistent with the change in the PW circulation and the accumulation of PW in the Arctic Ocean and specifically the Beaufort Sea since the 1990s. The black line in Figure 18 was obtained by adding the transports through the Fram and Davis Straits, across the Svalbard‐Severnaya Zemlya section and through Vilkitsky Strait (see Figure 2 for the positions of the sections).

### 4.4 Sources of Model Biases

Forcing imbalances, relaxation techniques, unresolved eddies, and missing processes in the mixed layer and upper part of the halocline, such as salt release from sea ice, all affect the models' performance. Here we only address the effects of the model resolution and accuracy of the model bathymetry. Higher‐resolution models better resolve narrow straits in the Canadian Arctic Archipelago. The experiments with the ORCA global model at 1°, 1/4°, and 1/12° horizontal resolution show that if the model configuration and forcing are the same, and the topography unchanged (i.e., the model topography is based on the real topography and not altered, like widening or closing straits and changing sill depth, etc.), higher‐resolution models simulate higher outflow west of Greenland and lower outflow east of Greenland. *Gerdes et al*. [2008] found that resolution is not the only parameter controlling the strength of the outflow through the Canadian straits, since a single, artificially widened channel in a coarse resolution model can result in a high outflow through the archipelago. Similarly to this study, *Jahn et al*. [2012] examined 10 widely used Arctic regional and global models and also found a substantial differences from model to model in simulating Arctic freshwater budgets. The models did not agree on the mean and variability of the liquid freshwater export though Fram Strait, although they did agree on the exports through the straits of the Canadian Arctic Archipelago. *Jahn et al.* [2012] concluded that the accurate simulation of the salinity variability is the principal improvement required to reduce the model differences and suggested that differences in the atmospheric reanalysis may be the source of the models' disagreement.

## 5 Summary and Conclusions

The dynamics of PW in the Arctic Ocean has been analyzed using model experiments with tracer releases in six coupled sea‐ice ocean models. In this intercomparison, we have analyzed PW pathways across the Arctic Ocean and through the Canadian Arctic Archipelago and Fram Strait and explained the drivers of the PW variability. In support of earlier proposed schematics of the PW circulation in the Arctic, the models simulated the two main pathways of PW. The eastward PW route follows the Alaskan shelf and then across the western Beaufort Sea and through the Canadian Arctic Archipelago and Baffin Bay in the Labrador Sea, while the western PW route leads along the Siberian shelf and then in the Transpolar Drift and through Fram Strait to the Nordic Sea and in the North Atlantic. The contribution of the pathways varied between models. In ICCMG, NAOSIM, and ORCA, there is a clear relationship between PW pathways and AO regimes: during the cyclonic years (1989–1996), the eastern pathway dominates, whereas during the anticyclonic years (1997–2007), the western one prevails. Other models do not show such a strong adherence to the AO circulation regimes.

Variations in the storage of the PW and associated FWC in the Arctic Ocean over the last 50 years have been examined using models and comparison with observations. There was a decrease in Pacific freshwater storage during the 1980s to 1990s and an increase during the last decade. Variations in FWC were similar to those of the PWC with a minimum in the 1990s. The simulated 1990s to 2009 anomalies are comparable with the freshwater inventory obtained from hydrography. The PW outflow through Fram and Davis Straits reflected the changes in PW in the Arctic Ocean, with a decrease in the PW export during last two decades. The model transit time for PW to cross the Arctic Ocean was estimated at 10–15 years, and the time for the PW outflow to reach a quasi‐equilibrium was ∼20 years. Sensitivity tests with ORCA were carried out to isolate the effects of model resolution, wind forcing, and Bering Strait on the dynamics of PW in the Arctic Ocean. In the ICMMG, NAOSIM and ORCA simulations, the Ekman convergence is a primary driver of the seasonal variability of PW in the Canadian Basin.

The most challenging issue of the present intercomparison study is that there are significant differences between the models and the observations, namely: (i) the models differ in their simulation of the upper ocean circulation in the Arctic Ocean and (ii) the models differ in simulating oceanic outflow into the North Atlantic, in particular disagreeing on the partitioning of the outflow between the Canadian straits and Fram Strait. The higher‐resolution models are expected to perform better in the Arctic where the Rossby deformation radius is small and the seafloor has complex small‐scale bathymetry. However, from the present study, it is not immediately obvious that such an improvement occurs. Moreover, a simplistic refinement of the model resolution in some cases paradoxically leads to stronger model biases, suggesting that model tuning for the high‐resolution models might be more challenging than for the lower‐resolution ones. In particular, the choice of mixing and advection schemes, lateral boundary conditions, and treatment of the oceanic bottom boundary layer have proven to be crucial for the simulations [*Penduff et al*., 2007]. Sensitivity tests exploring these model features will be beneficial for the tuning of models.

Another probable source of the divergence in model results is the forcing. Despite five out six models in this study being forced with NCEP reanalysis, the surface ocean circulation differs significantly. As sea ice dynamics moderates the atmospheric momentum transfer to the ocean, differences in sea ice cover in the models lead to different forcing of the ocean. Figure 18 gives an example of Ekman vertical velocity (pumping) averaged over the Canadian and Makarov Basins (Figure 2) following equations (B4a) and (B4b) in Appendix B in ICMMG, NAOSIM, and ORCA. From the plot, ICMMG has the weakest Ekman pumping, whereas in NAOSIM and ORCA, it is stronger. This explains why PWC variability is larger in these models. All three models show an increase of the Ekman downwelling between the 1990s and 2000s, consistent with the analysis of the observed winds [cf. *Yang*, 2009].

In this study, we have not discussed observational accuracy. Detection of the PW is based on the fine discrimination of chemical tracers which are not strictly conservative, but involved in the ecosystem. The nitrate/phosphate and Si techniques remain the only means to observe PW in the Arctic, and, as there are not independent methods, the accuracy of these techniques has some limitations, which need to be better quantified.

Model validation is a crucial part of the model development process. More data in the Arctic Ocean are now becoming available, including new data on ocean turbulence measurements, biogeochemical tracers, that are extensively used to identify water masses, and radionuclides to track the spread of the water masses. It is important for the ocean models to take advantage of the new observations and, vice versa, the observational campaigns could significantly benefit from robust high‐resolution model simulations.

## Appendix A Model Setup

### A1. Center for Climate System Research Ocean Component Model

The Center for Climate System Research Ocean Component Model (COCO) version 3.4 has been developed at the University of Tokyo [*Hasumi*, 2006; *Watanabe and Hasumi*, 2009]. The model domain contains the entire Arctic Ocean, Greenland Sea, and Baffin Bay. The model's spherical coordinate system is rotated so that the singular points were on the equator. The horizontal resolution is 1/4° × 1/4° (∼25 km) in the rotated coordinate system, and there are 28 model vertical levels. A sponge boundary condition is used at the southern boundary of model domain in the North Atlantic at 40°N. A seasonal cycle of the inflow through Bering Strait followed the mooring observations of *Woodgate et al*. [2005a], with an annual mean inflow of 0.8 Sv, and a seasonal maximum of 1.2 Sv in June and minimum of 0.4 Sv in December. The annual mean salinity of the Bering Strait inflow was set to 31.0.

The ocean used the uniformly third‐order polynomial interpolation algorithm (UTOPIA) [*Leonard et al*., 1995], the Gent‐McWilliams (*Leonard et al.* [1995]) eddy parameterization [*Gent and McWilliams*, 1990], and an isopycnal diffusion scheme for tracer advection and mixing. The sea ice component of the model used the zero‐layer thermodynamic formulation by *Semtner* [1976] and an elastic‐viscous‐plastic (hereafter EVP) rheology [*Hunke and Dukowicz*, 1997] for sea ice dynamics.

The atmospheric forcing components were constructed from the U.S. National Centers for Environmental Prediction/National Center for Atmospheric Research (NCEP/NCAR) reanalysis daily data set [*Kalnay et al*., 1996]. The model was also forced with monthly climatological river runoff [*Prange and Lohmann*, 2003, 2004].

The model was spun‐up from an ocean at rest with no sea ice for 10 years using a repeating annual cycle of the atmospheric forcing for 1979 from the initial temperature and salinity fields of the Polar Science Center Hydrographic Climatology (PHC) 3.0 database [*Steele et al*., 2001]. Following the spin‐up, the model ran from 1979 to 2008. During the spin‐up, temperature and salinity over the entire model domain were restored to the PHC 3.0 monthly mean. After the spin‐up was complete, the restoring was turned off, except for the marginal region at the southern model boundary in the North Atlantic. The model simulation for the period 1989–2007 was used for the present study (see Table 1 for details).

### A2. Estimating the Circulation and Climate of the Ocean, Phase II Model

The Estimating the Circulation and Climate of the Ocean, Phase II (ECCO2) model is an eddy‐permitting regional sea ice‐ocean numerical system based on the Massachusetts Institute of Technology general circulation model (MIT‐gcm) and optimized on observations [*Losch et al*., 2010]. The model is nested in the global outer sea ice‐ocean model. For this study (see Table 1 for details), a fully prognostic pan‐Arctic configuration was used with the same resolution as the global model, 18–23 km [*Nguyen et al*., 2011]. Boundary conditions at the lateral open boundaries at 55°N in the North Atlantic and the North Pacific were from the optimized global ECCO2 integration. No constraints were applied to the inflow through Bering Strait. It was fully prognostic.

For the vertical mixing, the ocean model uses the KPP scheme [*Pacanowski and Philander*, 1981] and the horizontal mixing was part of the advection. The oceanic tracer advection used the seventh order monotonicity preserving scheme [*Daru and Tenaud*, 2004] . The sea ice component used a viscous‐plastic (hereafter VP) rheology *Zhang and Hibler* [1997], ice thermodynamics for seven ice thickness categories with zero heat capacity, and a brine rejection parameterization [e.g., *Nguyen et al*., 2009].

Surface atmospheric boundary conditions (downward shortwave, downward longwave, wind, surface air temperature, and relative humidity) were from the Japanese 25 year ReAnalysis (JRA‐25) [*Onogi et al*., 2007]. Monthly mean estuarine fluxes of freshwater were based on the Regional, Electronic, Hydrographic Data Network for the Arctic Region (R‐ArcticNET) data set [*Lammers et al*., 2001]. Initial conditions for the ocean at the start of the integrations were from the World Ocean Atlas (WOA) 2005 [*Levitus et al*., 1998a, 1998b] merged with the Polar Science Center Hydrographic Climatology (PHC 3.0) data set [*Steele et al*., 2001]. The initial sea ice was from the Pan‐Arctic Ice Ocean Modeling and Assimilation System (PIOMAS) [*Schweiger et al*., 2011]. No restoring was applied to model temperature and salinity fields. The parameter space used in the ECCO2 regional Arctic solution was optimized using observations [*Nguyen et al*., 2011]. The model simulation for the period 1992–2007 with no spin‐up was used for the present study (Table 1).

### A3. The Institute of Computational Mathematics and Mathematical Geophysics Model

The Institute of Computational Mathematics and Mathematical Geophysics (ICMMG) regional sea ice‐ocean model has been developed in the Siberian Branch of the Russian Academy of Sciences [*Golubeva and Platov*, 2007]. The model domain includes the entire Arctic Ocean and the Atlantic Ocean north of 20°. The model grid is a spherical geographical grid in the North Atlantic with a horizontal resolution of 1° × 1°, merged with the displaced poles bipolar Arctic grid [*Murray*, 1996] along 65°N. As a result, the model horizontal resolution in the Arctic was ∼35 km. In the vertical, there are 35 model levels with the resolution decreasing with depth, and the highest resolution of 10 m near the surface. At the open lateral boundary in Bering Strait, a constant barotropic inflow of 0.8 Sv was prescribed. The radiation condition imposed on temperature and salinity at this boundary allowed for free advection of these fields out of the model domain, but when advection was inward, temperature and salinity were restored to the PHC 2.1. The prescribed Bering Strait inflow and the river discharge were compensated by transport through the outflow boundary at 20°S at each model time step.

The ocean component of the model used the Boussinesq hydrostatic approximation with a “rigid lid.” The mixed‐layer parameterization had a vertical adjustment based on the Richardson number. The temperature and salinity advection used the QUICKEST scheme [*Leonard*, 1991]. The ocean component is coupled with the CICE 3.14 sea ice model [*Hunke and Lipscomb*, 2006] with EVP sea ice rheology [*Hunke and Dukowicz*, 1997] and multicategory sea ice thermodynamics [*Bitz and Lipscomb*, 1999]. Sea ice advection utilizes a semi‐Lagrangian scheme [*Lipscomb and Hunke*, 2004].

The model was driven with daily atmospheric forcing from NCEP/NCAR reanalysis 1948–2012 [*Kalnay et al*., 1996] and by the monthly mean river runoff from the Global Monthly River Discharge Data Set [*Vorosmarty et al*., 1998] patched with the R‐ArcticNET data set [*Lammers et al*., 2001] in the Arctic Ocean.

The initial temperature and salinity data were adopted from the PHC 2.1 climatology [*Steele et al*., 2001]. Initially, the ocean was at rest and free of sea ice. There was no temperature or salinity restoring in the model domain except for the Gibraltar Strait mouth, where temperature and salinity were relaxed toward the PHC 2.1 [*Steele et al*., 2001] on a time scale of 180 days. The model simulation for the period 1989–2007 was used for the present study, with spin‐up 1948–1970 (Table 1).

### A4. North Atlantic/Arctic Ocean Sea Ice Model

A regional sea ice‐ocean North Atlantic/Arctic Ocean Sea Ice Model (NAOSIM) has been developed at the Alfred Wegener Institute for Polar and Marine Research. The model domain covered the Arctic Ocean, the Nordic Seas, and the Atlantic north of approximately 50°N (horizontal resolution was ∼28 km). The model configuration described here had 30 unevenly spaced levels in the vertical, with a 20 m‐thick level at the surface and level thicknesses gradually increasing with depth to 100 m. Open lateral boundary conditions in the Atlantic and in Bering Strait were formulated following *Stevens* [1991], allowing the outflow of model tracers (temperature, salinity, and passive tracers) and the radiation of waves [*Karcher et al*., 2012]. A net constant volume inflow of 0.8 Sv through Bering Strait was imposed.

The ocean component of the model is derived from the Geophysical Fluid Dynamics Laboratory modular ocean model MOM‐2 [*Pacanowski*, 1995] and includes a dynamic‐thermodynamic sea ice model with Hibler's VP rheology [*Hibler*, 1979].

The model was driven by daily atmospheric forcing 1948–2008 from NCEP/NCAR reanalysis [*Kalnay et al*., 1996]. A parametrization of river runoff used negative salt fluxes proportional to the seasonal climatology of runoff for each of the major Arctic rivers and was based on the study by *Prange and Lohmann* [2003, 2004]. The initial and open boundary oceanic conditions were taken from the PHC 2.1 climatology [*Steele et al*., 2001]. No restoring was applied to model temperature and salinity fields. The model simulation 1989–2007 was used for the present study, with spin‐up 1948–1988 (see Table 1).

### A5. Ocean Circulation and Climate Advanced Model

The Ocean Circulation and Climate Advanced Model (OCCAM) is a global z‐level model developed at the National Oceanography Centre [e.g., *Aksenov et al*., 2010, 2011].

The model has two separate grids joined along the Equator in the Atlantic Ocean and across the Bering Strait. The model horizontal resolution (∼8 km) is sufficiently fine to resolve eddies in most of the world ocean. In the central Arctic Ocean, the model is eddy resolving but eddy permitting at best on the continental shelf [e.g., *Aksenov et al*., 2011]. The model has 66 vertical levels with the resolution decreasing from 5 m at the surface to 40 m in the upper 400 m and to 200 m in the deeper ocean. Bering Strait is represented by a channel model connecting the Pacific and Arctic Oceans and the Bering Strait flow is fully prognostic.

The ocean model is based on the Geophysical Fluid Dynamics Laboratory Modular Ocean Model (MOM), described by, e.g., *Pacanowski* [1995] and coupled to a sea ice model with EVP rheology [*Hunke and Dukowicz*, 1997] and Semtner thermodynamics [*Semtner*, 1976]. The ocean model features a free surface formulation [*Killworth et al*., 1991], the Modified Split‐QUICK advection scheme [*Webb et al*., 1998] to advect tracers and sea ice and the KPP mixed‐layer model [*Large et al*., 1994]. On lateral boundaries, the no‐slip condition is applied.

The model was forced with the NCEP/NCAR 6‐hourly reanalysis atmospheric fields 1985–2007 [*Large et al*., 1997]. The simulations were initialized from rest using temperature and salinity data from the (WOA2005) merged with the PHC 2.1 data set in the Arctic [*Levitus*, 1998a, 1998b; *Steele et al*., 2001] and with sea ice and snow fields taken from the National Snow and Data Center (NSIDC) [*Comiso and Nishio*, 2008] and from the *Romanov* [1995] data sets. The river runoff was included in the restoring term.

The model sea surface salinity was relaxed to the monthly mean climatological values from WOA 2005 merged with PHC 2.1 data set [*Levitus et al*., 1998a, 1998b; *Steele et al*., 2001] with a time scale of 40 days. For this study we used simulations for the period 1989–2007, with spin‐up 1985–1988 (model details are in Table 1).

### A6. Nucleus for European Modeling of the Ocean Framework

The ORCA model configurations of the National Oceanography Centre Southampton has been developed within the Nucleus for European Modeling of the Ocean (NEMO) framework for ocean climate research and operational oceanography (http://www.nemo-ocean.eu/).

The ocean model is configured on a tripolar Arakawa C‐grid [*Arakawa*, 1966] with the model poles at the geographical South Pole, in Siberia and in the Canadian Arctic Archipelago. Due to the grid convergence, the model with nominal horizontal resolution of 1° has horizontal resolution of ∼111 km at the equator, increasing to ∼37 km in the Arctic Ocean. The model has 75 vertical levels with thicknesses of ∼1 m near the surface increasing to ∼204 m at 6000 m; there are 31 model levels in the upper 200 m. Bering Strait is resolved horizontally in the model with three grid nodes across the strait. In addition, an eddy‐admitting ORCA 1/4° (horizontal resolution of ∼10 km in the Arctic Ocean) and an eddy permitting/resolving 1/12° (horizontal resolution of ∼3 km in the Arctic Ocean) global configurations were used to test the effect of model resolution on the PW distribution and dynamics.

The model is a z‐level global sea ice‐ocean model and includes the ocean circulation model OPA9 [*Madec et al*., 1998]. The ocean model simulates vertical mixing using the turbulent kinetic energy mixing scheme (TKE) [e.g., *Blanke and Delecluse*, 1993] and uses the TVD advection scheme [e.g., *Madec et al*., 1998]. The ocean model is coupled to the Louvain‐la‐Neuve Ice Model sea ice model LIM2 [*Fichefet and Morales Maqueda*, 1997] with EVP sea ice rheology [*Hunke and Dukowicz*, 1997].

For the present study, the ORCA model was driven by the NCEP/NCAR Climate Ocean Reanalysis (CORE2) forcing data set [*Large and Yeager*, 2004]. Climatological monthly continental runoff was from *Dai and Trenberth* [2002]. The model was spun‐up from rest with the initial conditions for temperature and salinity derived from a monthly WOA 2005 climatology [*Levitus et al*., 1998a, 1998b], merged with the PHC 2.1 data set in the Arctic [*Steele et al*., 2001]. Initial sea ice conditions were from the ORCA25 integrations undertaken within the DRAKKAR high‐resolution modeling project [*Barnier et al*., 2006]. The sea surface salinity was relaxed toward the monthly mean climatological values from WOA 2005 *Levitus et al*. [1998a, 1998b] and PHC 3.0 on the time scale of 180 days. The setup used the GM eddy parameterization by *Gent and McWilliams* [1990] and employed isoneutral diffusion for tracer and horizontal bilaplacian viscosity for momentum [e.g., *Madec et al*., 1998]. The model simulation for the period 1989–2007 was used for the present study, with a spin‐up 1948–1988 (Table 1).

## Appendix B Diagnostics

Freshwater content, FWC, in the models was calculated referenced to the salinity *S*_*ref*_ = 34.8 and integrated through the whole water column and over the region in question:

(B1) $$\text{FWC}=\sum_{(i,j)\subset A}\sum_{k=1}^{k=K}\left(1-S/S_{ref}\right)\cdot \Delta z_{k}$$

Here *k* is the vertical index of model levels where salinity is ≤*S_ref_*, Δ*z_k_* is the thickness of the model level *k*, *S* is the model salinity, and (*i*, *j*) ⊂ *A* are the horizontal indices of the model cells contained in the selected region *A*.

Pacific Water content, PWC, in the models was calculated from the PW tracer fraction in each model cell PWT and integrated through the whole water column and over the region in question:

(B2) $$\text{PWC}=\sum_{(i,j)\subset A}\sum_{k=1}^{k=K}\text{PWT}\cdot \Delta z_{k}$$

here notations are as in equation (B1) above.

Referenced Pacific freshwater (PFW) fraction in the models was calculated from the PW tracer fraction PWT in each model cell referenced to the salinity *S_ref_* = 34.8 and PW mean salinity *S_pw_* = 32.0:

(B3) $$\text{PFW}=\text{PWT}\cdot \left(1-S_{pw}/S_{ref}\right)$$

Similarly to equation (B2), vertically integrated PFW content (PFWC) was obtained.

In the models, the stress curl, acting on the upper ocean interface (AKA curl of total surface stress) was a combined curl of wind stress on the open ocean surface, weighted by open water fraction (1 − *A_ice_*) and of stress at the sea ice‐ocean interface for sea ice‐covered areas, weighted by sea ice fraction (*A_ice_*) and was calculated as follows:

(B4a) $$Curl_{z}({\vec{\tau }}_{ocean})=\left(\frac{d\tau_{y}^{air/ocean}}{dx}-\frac{d\tau_{x}^{air/ocean}}{dy}\right)\cdot \left(1-A_{ice}\right)+\left(\frac{d\tau_{y}^{ice/ocean}}{dx}-\frac{d\tau_{x}^{ice/ocean}}{dy}\right)\cdot A_{ice}$$

and Ekman Pumping as

(B4b) $$EP=-Curl_{z}({\vec{\tau }}_{ocean})/(f\rho_{0})$$

here *x*, *y* are Cartesian coordinates, ${\vec{\tau }}_{ocean},\tau_{x,y}^{air/ocean},\tau_{x,y}^{ice/ocean}$ are the combined stress acting on the ocean interface, and *x*, *y* components of the stresses between air and ocean and ice and ocean respectively, *A_ice_* denotes the sea ice fraction, *f* is the Coriolis parameter, and *ρ*_0_ is the reference ocean density.
